# Adjuvanted HIV-1 vaccine promotes antibody-dependent phagocytic responses and protects against heterologous SHIV challenge

**DOI:** 10.1371/journal.ppat.1008764

**Published:** 2020-09-03

**Authors:** Kier Om, Dominic Paquin-Proulx, Maria Montero, Kristina Peachman, Xiaoying Shen, Lindsay Wieczorek, Zoltan Beck, Joshua A. Weiner, Dohoon Kim, Yifan Li, Thembi Mdluli, Zhanna Shubin, Christopher Bryant, Vishakha Sharma, Andrey Tokarev, Peter Dawson, Yohann White, Oliver Appelbe, Nichole R. Klatt, Sodsai Tovanabutra, Jacob D. Estes, Gary R. Matyas, Guido Ferrari, Carl R. Alving, Georgia D. Tomaras, Margaret E. Ackerman, Nelson L. Michael, Merlin L. Robb, Victoria Polonis, Morgane Rolland, Michael A. Eller, Mangala Rao, Diane L. Bolton

**Affiliations:** 1 US Military HIV Research Program, Walter Reed Army Institute of Research, Silver Spring, Maryland, United States of America; 2 Henry M. Jackson Foundation for the Advancement of Military Medicine, Bethesda, Maryland, United States of America; 3 Duke Human Vaccine Institute, Duke University School of Medicine, Durham, North Carolina, United States of America; 4 Thayer School of Engineering, Dartmouth College, Hanover, New Hampshire, United States of America; 5 EMMES, Rockville, Maryland, United States of America; 6 Department of Pharmaceutics, University of Washington, Seattle, Washington, United States of America; 7 AIDS and Cancer Virus Program, Frederick National Laboratory for Cancer Research, Leidos Biomedical Research, Inc., Frederick, Maryland, United States of America; University of Wisconsin, UNITED STATES

## Abstract

To augment HIV-1 pox-protein vaccine immunogenicity using a next generation adjuvant, a prime-boost strategy of recombinant modified vaccinia virus Ankara and multimeric Env gp145 was evaluated in macaques with either aluminum (alum) or a novel liposomal monophosphoryl lipid A (MPLA) formulation adsorbed to alum, ALFA. Binding antibody responses were robust and comparable between arms, while antibody-dependent neutrophil and monocyte phagocytotic responses were greatly enhanced by ALFA. Per-exposure vaccine efficacy against heterologous tier 2 SHIV mucosal challenge was 90% in ALFA-adjuvanted males (*P* = 0.002), while alum conferred no protection. Half of the ALFA-adjuvanted males remained uninfected after the full challenge series, which spanned seven months after the last vaccination. Antibody-dependent monocyte and neutrophil phagocytic responses both strongly correlated with protection. Significant sex differences in infection risk were observed, with much lower infection rates in females than males. In humans, MPLA-liposome-alum adjuvanted gp120 also increased HIV-1-specific phagocytic responses relative to alum. Thus, next-generation liposome-based adjuvants can drive vaccine elicited antibody effector activity towards potent phagocytic responses in both macaques and humans and these responses correlate with protection. Future protein vaccination strategies aiming to improve functional humoral responses may benefit from such adjuvants.

## Author summary

A clinically safe liposomal adjuvant can drive antibody effector activity towards increased phagocytic function relative to alum and this activity mediates protection against stringent mucosal SHIV infection in macaques.

## Introduction

Of the seven preventive HIV-1 vaccine trials conducted to date, only RV144 showed evidence of efficacy [1]. The rapid waning of protective immune responses and the reduction in vaccine efficacy from 60% to 31% at one and three years after initial vaccination, respectively, highlight opportunities to improve upon the RV144 pox-protein regimen [2, 3]. Increasing vaccine-elicited immune response magnitude, persistence, and functionality are active areas of investigation toward this end.

Numerous preclinical and clinical studies are exploring next generation adjuvants as a means to augment immunity. Historically, most licensed subunit vaccines have been adjuvanted with aluminum salts (alum). In recent years, promising alternatives to alum have emerged due largely to improved understanding of innate immunologic processes involved in pathogen recognition. As a result, multiple licensed vaccines now employ novel adjuvants for which robust safety profiles have been established, including the highly successful Shingrix shingles vaccine [4]. Promising results in many pre-clinical and clinical studies for other pathogens support their evaluation as a means to improve HIV-1 vaccine efficacy [5, 6]. As such, MF59, rather than alum, was used in the HVTN 702 phase 2b ALVAC-gp120 prime-boost clinical trial in the Republic of South Africa. This trial aimed to follow up on the RV144 trial but immunizations in the trial were recently halted. While the HVTN 702 futility finding limits the future prospects of this regimen due to manufacturing constraints, it raises key questions regarding whether parameters that differed between RV144 and HVTN 702 contributed to the disparate outcomes [7], such as the use of different adjuvants. Identification of adjuvants able to improve HIV-1 protein vaccine efficacy, even within the context of a pox-protein regimen, may aid in optimizing protein-based regimens.

Immune correlates analyses of RV144 and pre-clinical SHIV/SIV vaccine efficacy studies using unrelated vaccine regimens indicate potential mechanisms that mediate protection against mucosal HIV-1 transmission. Binding antibodies to the V1/V2 region of the envelope protein, IgG3 subclass V1/V2-specific antibodies, polyfunctional Env-specific CD4 T cells, low mucosal Env-specific IgA, and antibody-effector activities all show evidence of being associated with reduced risk of infection [2, 8–14]. Vaccine elicitation of HIV-1 broadly neutralizing responses capable of protecting against clinically relevant heterologous viral strains has proven elusive. As a result, there is growing interest in optimizing vaccine-induced humoral responses that exhibit non-neutralizing antibody-dependent effector activity. Development of robust assays that quantify non-neutralizing functional antibodies has further spurred investigations in this area [15, 16]. Moreover, Fc-mediated phagocytic functions are highly active in mucosal tissues [17], suggesting they may play an underappreciated role in protecting against HIV-1 and other mucosally transmitted pathogens. Vaccine strategies able to augment these responses have not been well described and could substantially improve efficacy [10, 11].

To evaluate a next generation adjuvant in combination with pox-protein HIV-1 vaccination against the most prevalent HIV-1 subtype, we immunized rhesus macaques with attenuated modified vaccinia virus Ankara (MVA) encoding HIV-1 *gag-pol* and *env* and a multimeric subtype C HIV-1 envelope protein, gp145 [18], adjuvanted with either alum or the Army Liposomal Formulation adsorbed to alum (ALFA) [19]. ALFA is believed to be an improvement to the highly successful but proprietary AS04 adjuvant, which contains free MPLA adsorbed to alum, and neither of these have been assessed for vaccination against HIV-1. Protection was determined by limiting-dose serial intrarectal challenge with the heterologous, tier 2, CCR5-tropic subtype C SHIV-1157ipd3N4 virus strain, considered to be a stringent model with limited vaccine efficacy achieved to date [20–22]. Adjuvanting with ALFA, but not alum, dramatically reduced the per-exposure infection risk, however, protection was limited to male animals. While most HIV-specific immune responses were similar between the adjuvant arms, antibody-dependent cellular (ADCP) and neutrophil phagocytic (ADNP) responses were augmented by ALFA. Evaluating for immunologic correlates of protection revealed ADCP, ADNP, and linear V2 peptide-specific rectal binding antibody responses as associated with protection. Further, a separate phase 1 study in humans demonstrated greater ADCP and ADNP responses upon gp120 vaccination adjuvanted with a related liposomal-MPLA-alum formulation relative to alum.

## Results

### Macaque vaccination and SHIV challenge outcome

**Fig 1:**
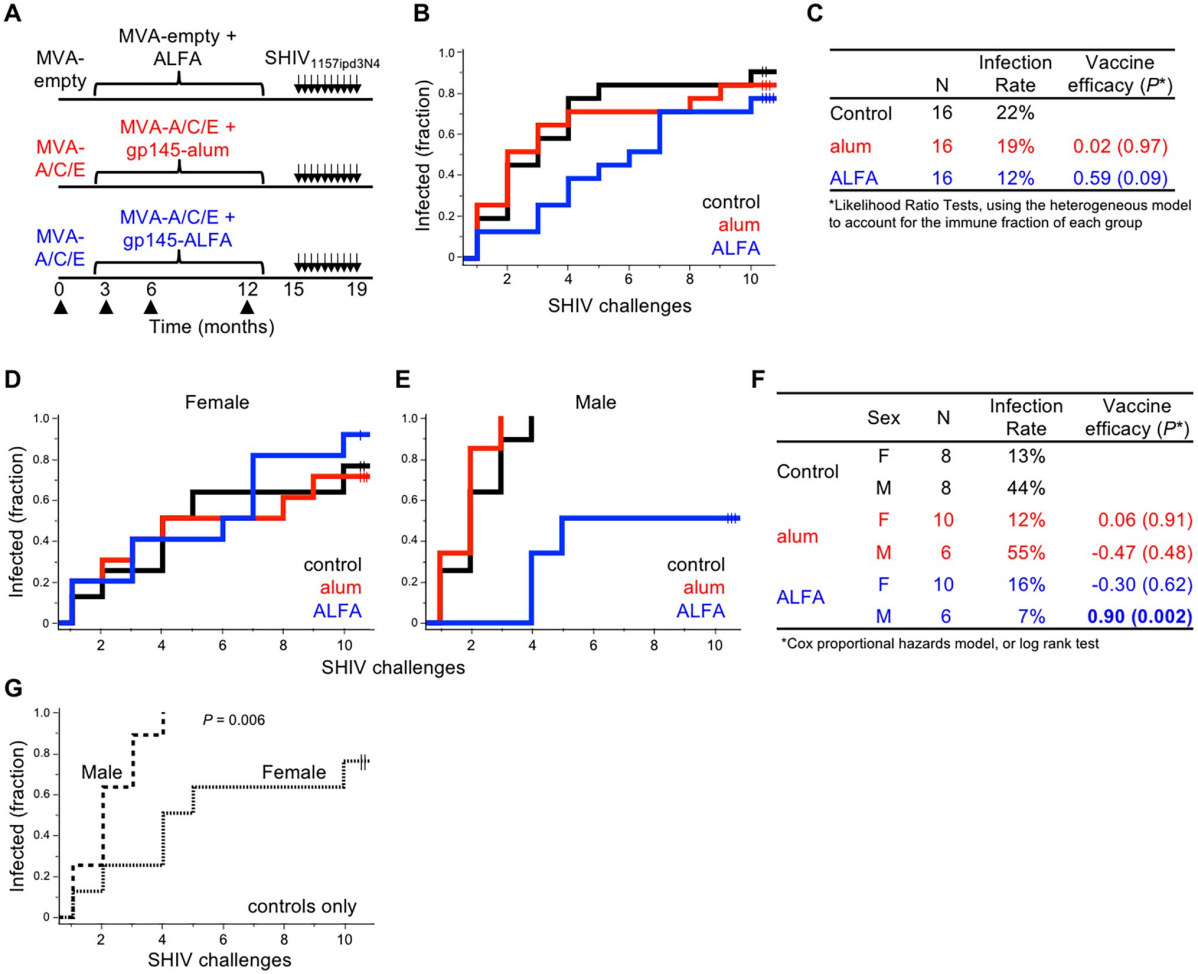
Study vaccination schedule and efficacy against challenge. **(A)** Rhesus macaques (n = 48) were vaccinated at the indicated time points (arrow heads) followed by up to ten low-dose intrarectal SHIV challenges (arrows). **(B)** Kaplan-Meier curves depict SHIV infection acquisition by vaccine group and **(C)** associated infection rates and vaccine efficacy. Animals that remained uninfected after ten challenges are indicated by tick marks. Infection rate is defined as the fraction of infections per total number of exposures. **(D-F)** Acquisition curves, infection rates, and vaccine efficacy by group are displayed separately for female and male animals. **(G)** Acquisition curves for control males (dashed) and females (dotted) are shown.

Forty-eight Indian-origin male and female rhesus macaques were enrolled in an HIV-1 vaccine efficacy study with SHIV challenge. Animals were manually assigned to three study groups minimized for differences in age, weight, sex, and *TRIM5* and *TRIMcyp* alleles. All animals lacked protective MHC class I alleles. Vaccine priming consisted of a multi-clade mixture of live-attenuated MVA encoding HIV-1 *gag*, *pol*, and *env* [23], followed by boosting with the MVA and a multimeric gp145 protein (CO6980v0c22, subtype C) at months three, six, and twelve (Fig 1A) [18]. Gp145 was adjuvanted with either aluminum hydroxide (AH, or “alum”) or Army Liposomal Formulation (ALF), which contains synthetic MPLA, adsorbed to AH (ALFA). Control animals received MVA lacking HIV-1 inserts and ALFA adjuvant alone.

Three months following the last immunization, serial limiting-dose intrarectal SHIV-1157ipd3N4 challenges were administered for up to ten exposures per animal (AID40). Animals were challenged simultaneously over two consecutive days or on a single day when fewer than 24 animals remained uninfected. The subtype C SHIV-1157ipd3N4 virus was selected as a stringent challenge strain for its tier 2 neutralization profile, heterologous sequence to the vaccine immunogens, and resistance to vaccine-mediated immunity [21, 22]. The sequence distance between gp145 and the challenge strain is comparable to that between two randomly selected sequences within an HIV-1 subtype. Time to infection was assessed by weekly plasma viremia measurements. Per-exposure infection rates were similar between the alum and control arms (19% and 22%, respectively), while ALFA reduced the per-exposure infection risk by 59% compared to controls (*P =* 0.09, 12% infection rate, Fig 1B and 1C). Nine animals from all three groups resisted all ten prespecified challenges. Subsequent challenge with a five-fold higher dose resulted in infection of all but one animal (alum group) after three challenges, indicating susceptibility to infection across the cohort.

While the study was not designed to detect differences between sexes, sex can influence both vaccine-elicited immune responses as well as vaccine efficacy [24, 25], and the majority of macaque HIV-1 vaccine trials have historically been performed in male animals. In order to account for potential differences in the risk of infection, the analysis of vaccine efficacy was stratified by sex. Remarkably, ALFA-adjuvanted vaccine efficacy was highly sex-specific, with protection exclusively in male animals (90% efficacy, *P =* 0.002, Fig 1D–1F). Males in the control (n = 8) and alum (n = 6) groups were all infected by the fourth challenge, while all ALFA males (n = 6) resisted the first three challenges and 50% remained uninfected following all ten challenges, which extended to seven months after the last vaccination. Thus, among males, the control and alum arms were highly susceptible to infection while ALFA afforded substantial protection both short- and long-term. In contrast, no efficacy was observed with ALFA or alum among females.

Of note, SHIV acquisition was delayed in control females compared to control males (infection rates per-exposure of 13% and 44%, respectively, *P =* 0.006, Fig 1F and 1G). Infection rates were also significantly higher in males than females in the alum arm (*P =* 0.005). The increased resistance to infection among females may reflect sex-specific innate barriers to infection, as has been observed in other settings [26, 27]. Markers of inflammation and gastrointestinal tract integrity, such as type I interferon response (Mx1) and gastrointestinal neutrophil infiltration (MPO), in rectal lamina propria prior to challenge did not differ between sexes across or within study arms (S1A Fig). To determine whether the rectal mucosa microbiome influenced infection rate, beta diversity statistical metrics were used to compare overall microbial compositions. Microbial composition differed between males and females using both Bray Curtis and weighted Unifrac analyses (*P =* 0.005 and *P =* 0.007, respectively), with significant differences observed at the Family level (S1B Fig), consistent with previously described sex-based differences [28]. However, no significant associations with time to infection were observed in either sex, among all animals or vaccinated only.

Viral replication post-infection did not differ between vaccinated and control animals: peak and set point viremia were comparable across groups (S2A and S2B Fig). These and similar findings in other pox-protein clinical and pre-clinical vaccine studies are consistent with limited magnitude and potency of cytotoxic T cell responses elicited by these regimens [1, 29, 30]. A sex-based difference in viremia was observed, however, within the ALFA group, as set point viremia was higher among ALFA-vaccinated females than males (*P =* 0.04 (S2C Fig)). Females are known to produce more IFN-α in response to Toll-like receptor 7 (TLR7) stimulation (e.g. challenge virus genome) than males [27, 31]. However, viral sequencing of two control female animals (with relatively high viral load) during acute infection did not reveal any selection barrier for type I interferon-resistant virus among females (S2D Fig).

### Vaccine humoral immunogenicity

**Fig 2:**
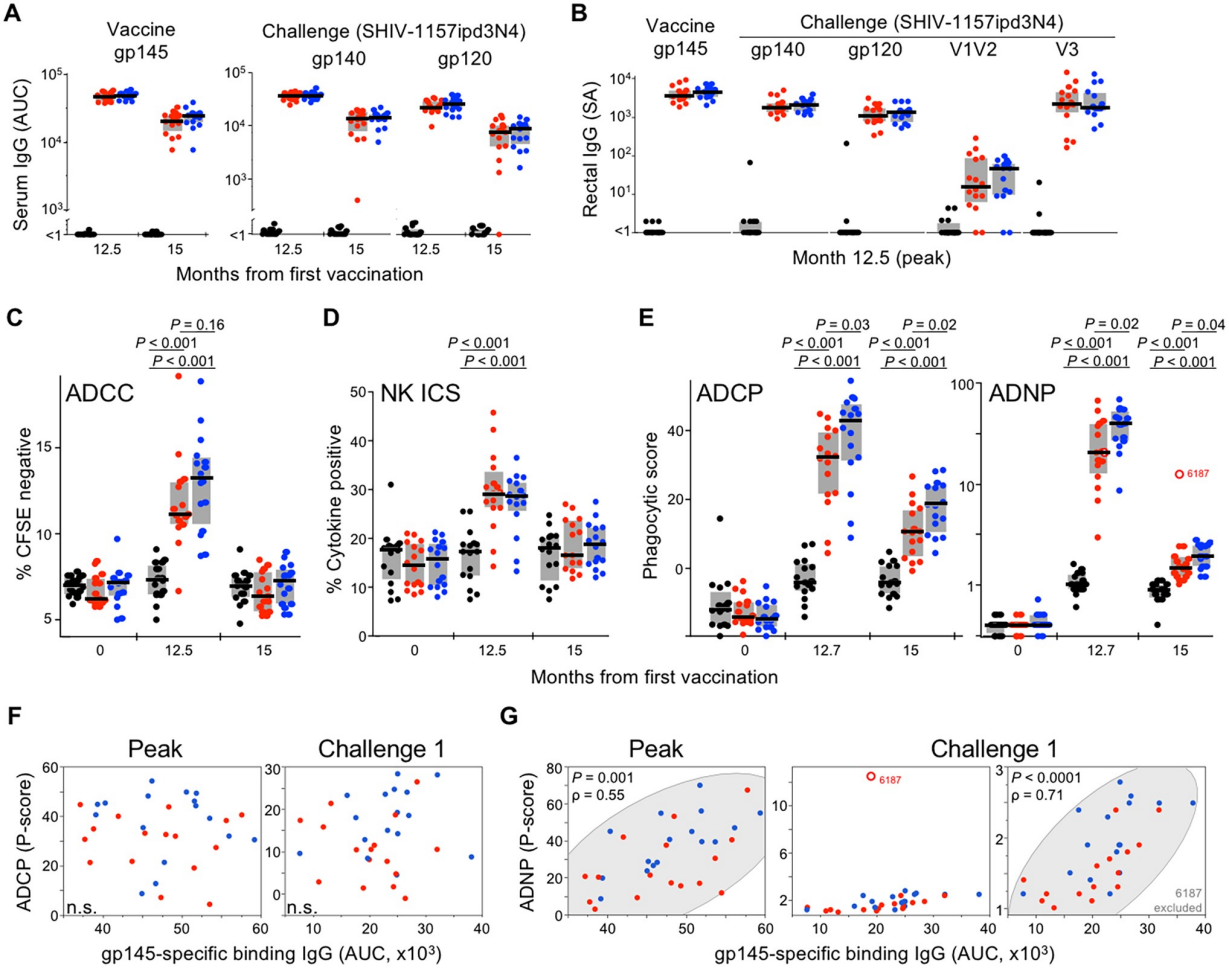
Env-specific antibody binding and effector responses elicited by vaccination. **(A)** Serum IgG binding antibody responses to gp145 and SHIV-1157ipd3N4 antigens are shown at peak and time of challenge. Area under the curve (AUC) reflects BAMA MFI values across serially diluted serum. **(B)** Peak rectal binding IgG responses reported as specific activity (SA), calculated as MFI * dilution / IgG concentration. All active arm responses in (A-B) are significantly different from the control arm, but not from each other. **(C)** Longitudinal serum ADCC responses were assessed by antibody-dependent lysis of CFSE-labeled Env-coated target cells and are reported as an increase in CFSE-negative target cells. **(D)** Antibody-dependent NK cell responses were measured by Env stimulation in the presence of animal sera followed by intracellular cytokine staining (ICS) for IFNγ, TNFα, and MIP1β. The frequency of NK cells expressing one or more cytokine Boolean combinations is shown. **(E)** ADCP and ADNP responses were assessed using THP-1 monocyte and human neutrophil effectors, respectively. ADNP open symbol depicts animal that resisted three additional high-dose challenges. Effector assays were performed with CO6980v0c22 multimeric gp145. For ADCP and ADNP, serum was used at month 0 and plasma at months 12.7 and 15 due to sample availability. Gray bars reflect interquartile range; black lines depict medians; *P* values in (C-E) reflect Wilcoxon rank-sum test. **(F-G)** Spearman correlation between ADCP and ADNP responses and serum gp145-specific binding antibodies at indicated time points. Symbol colors reflect control (black), alum (red), and ALFA (blue) groups.

Binding, neutralization, and non-neutralizing functional antibody responses to multiple antigens were assessed in serum and rectal secretions. Remarkable serum IgG responses to HIV-1 Env gp140, gp120, gp70-scaffolded and cyclic V1V2 and V3 antigens, gp41, and the gp145 immunogen were observed by ELISA, multiplex Luminex-based assays (BAMA), and Fc array analysis in both active arms (Fig 2A, S3–S5 Figs). These included antigens derived from the vaccine, challenge, and unrelated strains across multiples subtypes. However, response rates and magnitude were similar between the alum and ALFA arms at all time points assessed, including following each vaccination as well as the time of challenge. Hierarchical clustering and principal component analysis of the binding responses assessed by Fc array did not discriminate between active arms and no individual component responses were significant after FDR-adjustment (S4 Fig). Env-specific rectal mucosa IgG responses were also similar in magnitude between arms, including to linear peptides from multiple HIV-1 strains, while minimal IgA responses were observed (Fig 2B, S5 and S6 Figs). Specific activity to both the vaccine and challenge Envs ranged from 10^3^−10^4^, with 100% response rates. Binding antibody responses to infected cells were also robust but again did not differ (S3F Fig). Sex-specific analyses revealed greater binding responses to some antigens among females that received ALFA adjuvant compared to alum, but these differences were inconsistent across time points and did not withstand adjustment for multiple comparisons.

Neutralizing antibody responses to multiple tier 1 pseudoviruses were achieved and boosted with each immunization (S7 Fig). No tier 2 neutralizing responses were observed, including to the matched vaccine and SHIV-1157ipd3N4 challenge pseudoviruses. Response rate and magnitude were generally similar between the alum and ALFA arms, as were neutralization breadth and potency scores. Taken together, while strong binding and tier 1 neutralizing antibody responses were elicited by the MVA-gp145 regimen, adjuvanting with ALFA did not substantially increase these responses relative to alum.

The importance of antibody effector activities in controlling pathogen replication is increasingly appreciated in large part due to their identification as immune correlates of vaccine-reduced infection risk [9–11, 25]. To determine the extent of cell-mediated antibody effector responses, we assessed antibody-dependent cell-mediated cytotoxicity (ADCC), ADCP, ADNP, and antibody-dependent natural killer (NK) intracellular cytokine staining (ICS) responses at peak immunogenicity and at the time of challenge. ADCC, ADCP, ADNP and NK ICS responses were elicited in both active arms (Fig 2C–2E). ADCC and NK ICS responses were similar between the alum and ALFA groups at peak (*P =* 0.16, *P* = 0.6, respectively) and waned to baseline levels by the time of challenge. ADCP and ADNP, by contrast, were robust at peak, sustained for several months, and consistently greater with ALFA than alum (Fig 2E). Of note, the animal that resisted all ten prespecified challenges as well as the high-dose supplemental challenges maintained exceptional ADNP responses at the time of challenge. The increased ADCP activity by ALFA relative to alum was most apparent within males, and ADNP within females, though trends were clear in both sexes (S8 Fig). Results obtained with human and rhesus neutrophil effectors were correlated. ADNP, but not ADCP, responses correlated with concurrent binding antibody titers (Fig 2F and 2G), suggesting that ALFA specifically increased monocyte phagocytic antibody effector activity independent of binding antibody levels.

### Vaccine cellular immunogenicity

**Fig 3:**
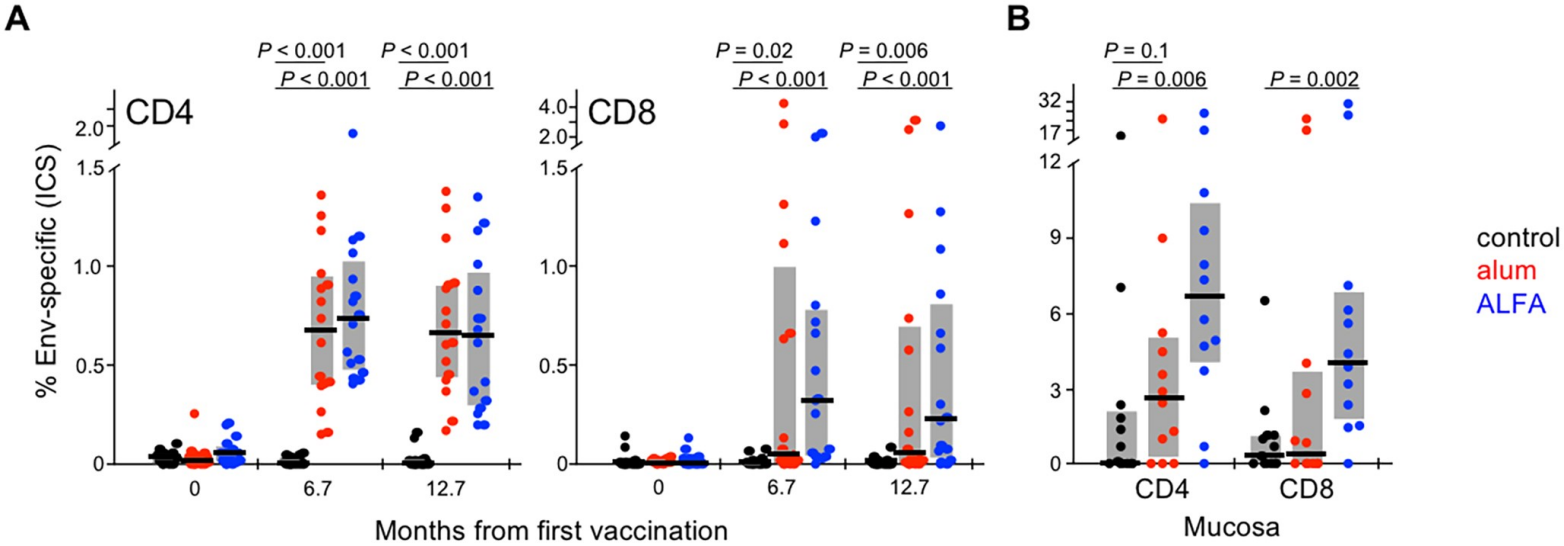
HIV-specific CD4 and CD8 T cell responses. **(A)** PBMC HIV-1 Env-specific CD4 and CD8 T cells were measured by ICS for IFNγ, TNFα, IL-2, and CD154 three weeks following the third (month 6.7) and fourth (month 12.7) immunizations. The frequency of memory cells that expressed one or more cytokines following *ex vivo* Env PTE peptide pool stimulation is shown for each animal by arm. CD4 responses represent summation of responses to pools 1–3 (spanning amino acids 4–585), while CD8 are to pool 3 only (amino acids 413–585). **(B)** Bronchoalveolar lavage Env-specific CD4 and CD8 T cells were measured by ICS four weeks post-infection for animals infected by challenges one through ten. Gray bars depict interquartile range; black lines depict medians; *P* values reflect Wilcoxon rank-sum test.

Pox-protein vaccination elicits modest but potent CD4 T cell immunity and polyfunctional CD4 T cell responses correlated with reduced infection risk in RV144 [8]. To assess the induction of antigen-specific T cell responses in MVA-gp145 vaccinated animals, we performed T cell ICS on peripheral blood and mucosal mononuclear cells. PBMC Env-specific CD4 T cell responses rates were uniformly high following the third and fourth vaccinations in both the alum and ALFA arms, with all animals responding (Fig 3A). The frequency of Env-specific CD4 T cells ranged from 0.2–1.7% of the memory compartment and did not differ between adjuvants. CD4 response quality, as assessed by either the Boolean combinations of cytokines expressed by Env-specific cells using SPICE or the COMPASS functionality and polyfunctionality scores, was similar between the adjuvant arms (S9 Fig). CD154 single-positive T cell responses predominated. CD8 T cell responses to Env were more variable, observed in approximately half of the animals in each arm, and restricted to just one of the three Env peptide pools assessed. Among responding animals, the magnitude was robust, at 0.2–4% of memory CD8 T cells. Again, there was no difference between the alum and ALFA vaccinated groups. The MVA HIV *gag-pol* insert did not show evidence of priming SIV Gag cross-reactive responses, as SIV Gag-specific CD4 and CD8 T cell responses were not apparent in the active arms post-vaccination and comparable between the control and active arms post-infection. Vaccine elicited CD8 T cell responses were not associated with reduced viral load at peak or setpoint.

To assess adaptive mucosal T cell responses, ICS was performed on bronchoalveolar lavage (BAL), an accessible extra-lymphoid mucosal effector site with less background than rectal samples, four weeks post-infection. Responses early after infection in vaccinated animals represent an anamnestic response primed by vaccination and are thus expected to exceed the primary response seen in control animals. As mucosal samples are prone to more ICS background than PBMC and background values are often within the range of vaccine-elicited responses, probing the more vigorous anamnestic post-infection response can be a useful approach for assessing vaccine priming of cellular responses. Indeed, acute infection HIV-specific BAL CD4 and CD8 T cell responses were much greater in ALFA vaccinees than controls (Fig 3B). Median CD4 and CD8 T cell responses with ALFA were ~6% and 4%, respectively, versus <1% for both subsets in control animals. These anamnestic responses were less pronounced with alum (3% and 0.5%), which did not differ from controls, suggesting more effective mucosal T cell priming by ALFA than alum.

### Immune correlates of protection

**Fig 4:**
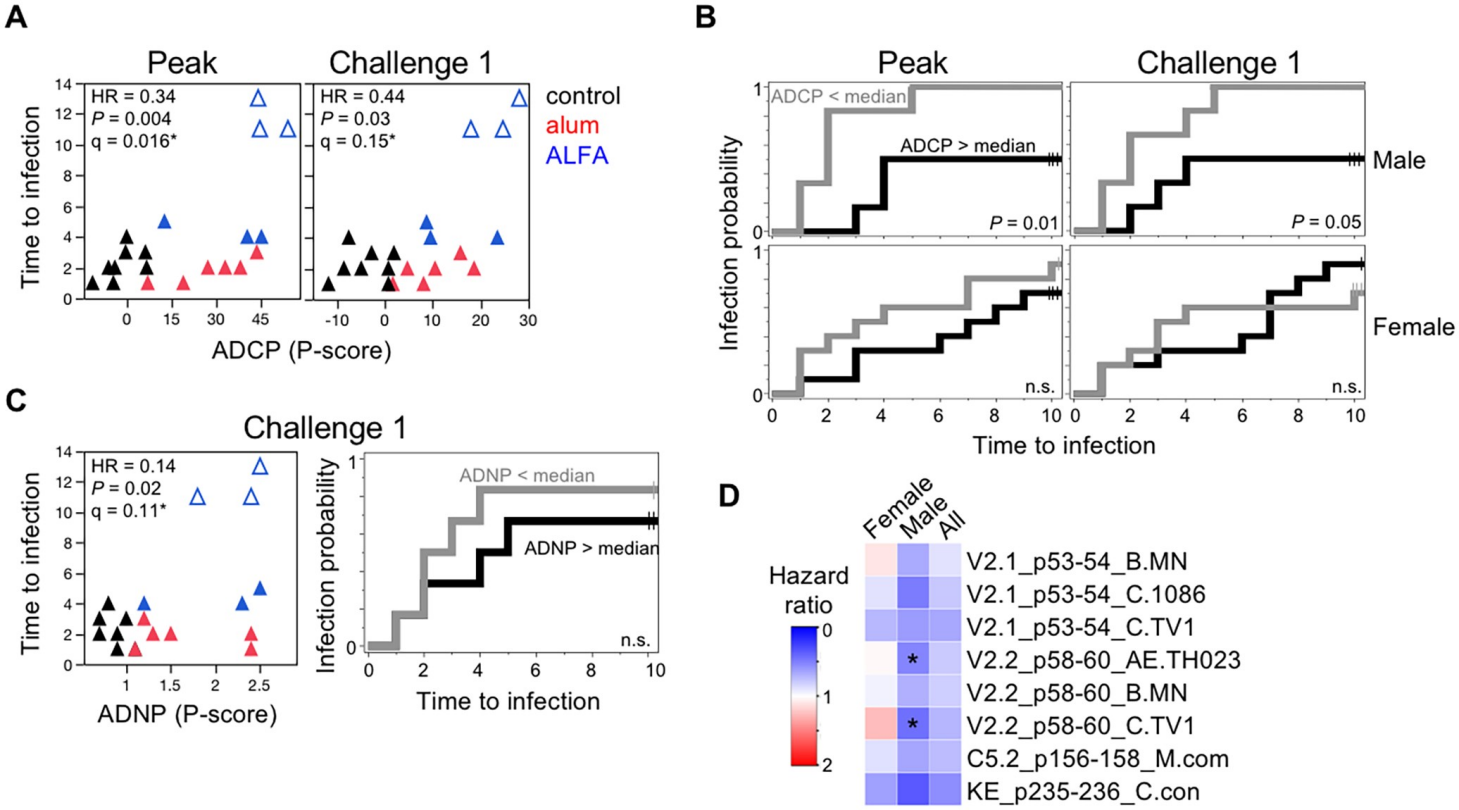
Vaccine-mediated immunologic correlates of protection. **(A)** ADCP phagocytic (P)-scores are plotted against the challenge number that resulted in SHIV infection for male animals. Peak (month 12.5) and time of challenge (month 15) responses are shown. Unadjusted (*P*)- and FDR-adjusted (q)-values reflect significance test of the continuous effects of the responses on the risk of infection (*, q<0.2). Animals that resisted the ten prespecified challenges are depicted as open symbols. HR, hazard ratio. **(B)** Kaplan-Meier infection probabilities by ADCP responses above (gray) or below (black) the median at peak immunogenicity (left) and time of challenge (right) are shown for vaccinated animals by sex. Log-rank test p-values comparing high vs. low responders are indicated. **(C)** Time of challenge ADNP responses are plotted against the infecting challenge for male animals. FDR adjustment included all in-class assays (S3 Table), as the human neutrophil phagocytic assay was filtered out of the primary analysis due to correlation with the rhesus ADNP assay (S1 Table). Kaplan-Meier infection probability by ADNP responses above and below the median at the time of challenge are shown for male vaccinated animals. **(D)** Heatmap depicts association between peak rectal IgG Env epitope-specific responses and infection risk. Hazard ratios for the peptide array epitopes that remained in the analysis after filtering out highly correlated probes and exhibited protective trends in males (*P*<0.2) are shown, separately by sex and in all animals (*, *P*<0.05, q>0.2).

To identify potential vaccine-mediated immune correlates of protection, the numerous humoral and cellular immunogenicity measurements were first filtered to remove highly correlated assays (S1 Table). Assays were assessed for association with the risk of infection via Cox proportional hazards models, with sex and vaccine arm included as covariates. ADCP responses to gp145 were significantly protective in males at both peak (HR = 0.34, *P* = 0.004, q = 0.016) and time of challenge (HR = 0.44, *P =* 0.03, q = 0.15, Fig 4A, S2 Table). Eighty percent of vaccinated animals with peak ADCP responses below the median were infected after just two challenges, while animals above the median remained uninfected during this time and either succumbed to subsequent challenges or resisted all prespecified challenges (Fig 4B). In the unfiltered analysis, ADNP responses assessed at the time of challenge were also protective in males (HR = 0.14, *P =* 0.02, q = 0.11, Fig 4C, S3 Table). These results were not affected by outliers or non-proportional hazards; similar estimates were seen in the other sensitivity analyses in vaccinated animals only, including when not accounting for the vaccine arm (S4 and S5 Tables). The effect was observed even after adjustment for vaccine regimen, indicating responses were protective across study arms. Though ADCP and ADNP responses were more strongly induced by ALFA than alum (Fig 2E), similar hazard ratios were observed with and without adjustment for study arm, indicating protective effects that were conserved across groups.

A protective signal trend was also identified among rectal IgG peptide microarray responses. Binding antibody responses to two V2 epitopes exhibited evidence of a protective effect among males (*P*<0.05, q>0.2, Fig 4D, S10 Fig, and S2 Table), while weaker trends were observed for several other V2 epitopes (*P*<0.2). Sensitivity analysis at the peptide level showed similar protective responses. These responses did not appear to be consistently elicited by either vaccine regimen, however. Rectal IgA BAMA responses to several antigens were also protective (S2 and S3 Tables), though these results were driven primarily by the three uninfected males. No significant correlates of protection were identified in females. Penalized multivariate immune correlates of risk analysis did not reveal compelling protective multi-assay responses (S6 Table).

There was little evidence of associations between immune responses at either peak or time of challenge and peak viremia among the animals infected within ten challenges. CD4 T cell Env-specific ICS responses at time of challenge were significantly associated with lower log-2 transformed peak viral load (~50% decrease in mean viremia with each SD increase, *P =* 0.03, q = 0.13).

### Human antibody-dependent phagocytic responses to liposomal-MPLA adjuvanted HIV-1 vaccine

**Fig 5:**
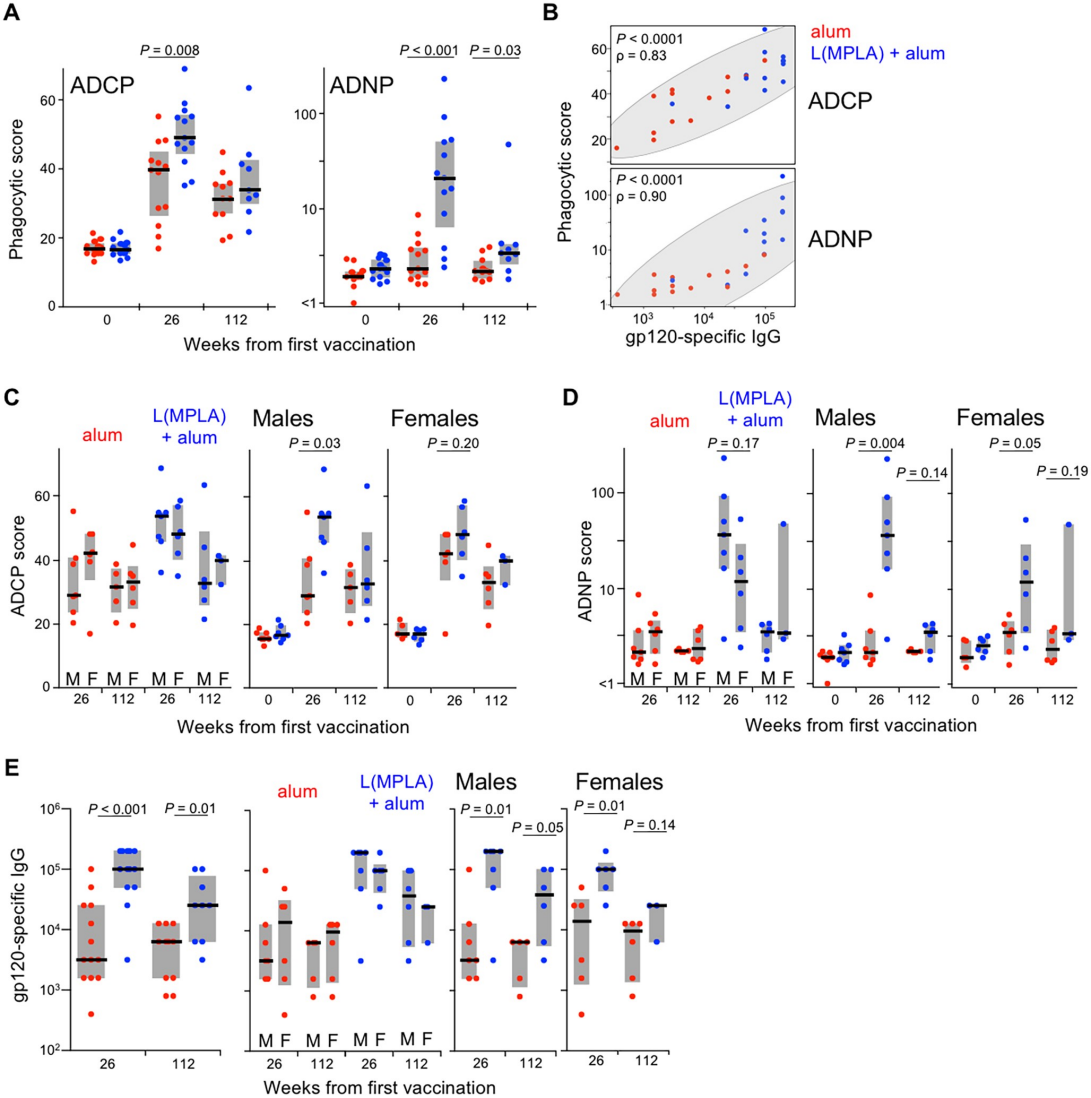
Env-specific antibody-dependent phagocytic responses elicited by liposomal-MPLA-alum adjuvant in humans. **(A)** Serum ADCP and ADNP responses in humans vaccinated at weeks 0, 8, 24, and 72 with HIV-1_SF-2_ gp120 protein adjuvanted with either alum (red) or liposomal-MPL adsorbed to alum (blue) were assessed at weeks 0 (n = 12 and 13, respectively), 26 (n = 13 each arm), and 112 (n = 11 and 9, respectively). **(B)** Week 26 ADCP and ADNP responses are plotted against week 26 gp120-specific binding IgG endpoint titers with Spearman’s rho and *P* values indicated. **(C)** ADCP and **(D)** ADNP responses are stratified by participant sex to compare responses within each adjuvant study arm (left; M, male; F, female) or between arms within each sex (right). **(E)** gp120_IIIB_ Env-specific binding IgG endpoint ELISA titers are shown for all participants combined (left), by sex within each vaccine arm (middle), and by arm within each sex (right). Gray bars depict interquartile range; black lines depict medians; *P* values reflect Wilcoxon rank-sum test.

To determine whether adjuvanting with liposomal-MPLA also increases antibody-dependent phagocytic activity in humans, we analyzed responses in 26 participants of a phase 1 clinical trial (AVEG015, NCT00001042) that compared HIV-1_SF-2_ gp120 protein vaccination adjuvanted with either alum or a liposomal-MPLA-alum formulation (n = 13 per arm) [32]. The liposome components used in this study were identical to those of ALFA, but the protein immunogen was encapsulated within the liposomes rather than external to them [33]. Protein immunizations were administered at weeks 0, 8, 24, and 72. Prior analysis of Env-specific IgG binding antibody responses revealed 32- and 4-fold higher median titers with the liposomal adjuvant compared to alum at peak immunogenicity (week 26) and week 112, respectively [32]. Here, we found peak ADCP and ADNP responses were also greater (Fig 5A, *P =* 0.008 and *P*<0.001, respectively). Moreover, alum only weakly elicited ADNP responses, which marginally increased from baseline to week 26 (*P =* 0.07). In contrast, much higher ADNP responses were generated with the liposomal adjuvant (*P*<0.001). Of note, the magnitude of the increase in peak ADCP responses relative to alum was similar to that observed in the macaques: the median score increased by about ten in both liposomal groups; ADNP scores improved by a larger margin in macaques (~20 versus 40 in humans and NHP, respectively). At week 112, ADCP responses were still apparent but did not differ by adjuvant, while ADNP responses persisted in the liposome group (*P =* 0.01 relative to baseline) and remained greater with liposome than alum (*P =* 0.03). Peak ADCP and ADNP responses were both highly correlated with contemporaneous Env-specific binding antibody titers (Fig 5B), suggesting that the increase in peak phagocytic responses achieved by liposomal adjuvanting is most likely explained by greater titers.

Since AVEG015 study participants were a mix of males and females, we analysed responses stratified by sex. Neither ADCP, ADNP, nor binding IgG responses differed between males and females within each adjuvant arm (Fig 5C–5E). However, the increase in peak phagocytic responses by liposome was more prominent among males, with greater ADCP (*P* = 0.03) and ADNP (*P* = 0.004) relative to alum. In contrast, weaker gains were observed among females (*P* = 0.20 and *P* = 0.05, respectively). Both males and females exhibited increased Env-specific binding IgG responses with the liposomal adjuvant (Fig 5E). Taken together, liposome increased binding responses in both sexes, while males benefited more than females for ADCP and ADNP functional responses.

## Discussion

HIV-1 Env gp145 protein vaccination adjuvanted with ALFA combined with MVA priming demonstrated a remarkable 90% efficacy in male macaques against a stringent, difficult to neutralize tier 2 heterologous SHIV challenge, while adjuvanting with alum conferred no protection. Few pre-clinical HIV-1 vaccine studies have achieved this level of protection. ALFA increased HIV-specific ADCP, ADNP and mucosal T cell responses, and the antibody-dependent phagocytic responses highly correlated with reduced risk of infection. While vaccine-mediated protection was not observed in females, immune responses were largely similar in males and females and therefore the ALFA adjuvant appeared equally immunogenic in both sexes. Evaluation of liposomal-MPLA-alum adjuvanted gp120 in humans also demonstrated increased Env-specific ADCP and ADNP responses relative to alum. These data support future studies with ALFA as a promising and safe adjuvant formulation that elicits protective antibody effector responses in both NHP and humans.

Our pre-clinical vaccine efficacy study was modeled after the RV144 trial, as was the HVTN 702 trial. Despite the failure of the latter to demonstrate a reduction in infection rates, there is compelling evidence that the results from RV144 remain valid. These clinical trials differed in many respects, including vaccine immunogens, adjuvant, HIV-1 circulating strains and their diversity, and infection risk within the respective populations [7]. In addition, the hypotheses generated by RV144 regarding the protective role of V1V2-specific antibody responses have been borne out repeatedly in several macaque efficacy studies [34], including here. The MF59 adjuvant employed in HVTN702 was previously reported to increase both binding and non-neutralizing antibody responses relative to alum in macaques [35], and therefore it will be important to determine whether these responses were also augmented in humans. Though the present macaque study evaluated adjuvanting of protein vaccination in the setting of pox vector priming, our findings are expected to be relevant for other regimens as well. ALFA increased antibody-dependent phagocytosis in the absence of pox vector priming in the AVEG015 clinical trial, indicating adjuvant-mediated augmentation of ADCP and ADNP responses is not limited to pox-protein vaccination.

Our finding that antibody-dependent phagocytic activities correlate with vaccine efficacy lends substantial support to the emerging notion that non-neutralizing functions of antibodies can mediate protection from HIV/SIV infection with multiple vaccine regimens [9–12]. Moreover, we demonstrate that liposomal adjuvants can be used to increase ADCP and ADNP responses in both NHP and humans relative to alum. ADCP might be a particularly relevant mechanism of protection for intrarectal transmission, where macrophages greatly outnumber NK cells and neutrophils [17]. Previously, the adjuvant MF59 elicited greater antibody effector functions than alum but did not result in protective immunity [35]. Interestingly, we found that in NHP, ADCP was not associated with IgG binding titers while ADNP was, suggesting that distinct pathways might be involved in the development of these effector functions. Addition of a TLR4 agonist to alum in a prior study did not significantly increase ADCP responses [36], possibly implicating the liposomes or liposomes in combination with MPLA for increased responses observed with ALFA. Incorporating different TLR agonists into liposomal adjuvants has also been shown to influence antibody effector functions: TLR4 plus TLR7 agonists resulted in higher ADNP and ADCC responses than TLR4 agonist plus QS21, while the latter promoted antibody-dependent complement deposition and distinct antibody glycoforms [37]. Given the importance of IgG glycosylation in mediating phagocytic effector activity [15], it is plausible that alum and ALFA elicit distinct glycosylation patterns that impact phagocytic responses. Alternative possibilities include antibody subclass, epitope specificity, and valency of immune complexes. Liposomal adjuvants may also increase immune responses by promoting uptake of adjuvant-antigen complexes by antigen presenting cells [38]. Future studies evaluating ADCP and ADNP responses elicited by this adjuvant family should consider additional assays that incorporate native conformations of Env, such as virion- or infected cell-based assays, as well as species-matched effector cells to account for Fc receptor differences, as they may better reflect antibody effector activity *in vivo*. The Env-coated bead-based assays used here generally compare favorably to virion phagocytosis assays and have revealed immune correlates identified in other vaccine studies, but they may poorly recapitulate aspects of infected cell phagocytosis.

While liposomal-MPLA-alum adjuvant increased antibody-dependent phagocytic responses in both humans and NHP, correlations with binding titers differed between the studies, possibly due to differences in the adjuvant formulations. In AVEG015, protein was encapsulated within the liposomes [32, 33], while ALFA used in the NHP study consisted of immunogen adsorbed to the aluminum salt rather than inside the liposome [19]. Antigen physically associated or attached to the outer surface of liposomes can elicit superior binding and neutralizing antibodies compared to liposome-encapsulated antigen [39]. Other features of ALFA that may be relevant include the use of unilamellar rather than multilamellar liposomes, a lower phospholipid:lipid A ratio, and less MPLA.

Non-neutralizing antibodies have also been associated with control of SIV replication after vaccination [37, 40], and yet we did not observe any effect of vaccination on viral load peak or set point. The challenge virus may partially explain these conflicting observations, as post-acute viremia is typically less robust with SHIV. Our findings are consistent with the RV144 trial, however, in which viral load did not differ between the vaccine and placebo groups [30].

It is not clear why vaccine efficacy was observed in male but not female macaques. One possibility is that the males provided a more sensitive model for detecting adaptive immune-mediated reductions in infection risk given their increased susceptibility to infection. Of note, most pre-clinical macaque vaccine efficacy studies are conducted exclusively in males or with insufficient power to stratify outcome by sex, and thus opportunities to assess sex-based differences have been limited. One study using recombinant adenovirus priming and Env protein boosting observed a delay in SIV acquisition following intrarectal challenge in female but not male macaques [41]. While the opposite sex was protected in that study compared to the current study, these findings speak to the existence of poorly understood sexual dimorphisms in primate immunity, which is well known in humans and an area of active investigation [26, 42]. Future well-powered studies including both sexes will be important to assess the influence of sex on vaccine outcomes. Our observation in humans of humoral responses in males benefiting more than females from the liposomal adjuvant relative to alum further supports sex-based differences in adaptive immune responses to adjuvanted vaccines.

The sex dichotomy in SHIV acquisition rate observed in this study was also unexpected and striking. Intrarectal infection risk was considerably higher in control males than control females (*P =* 0.006). To our knowledge, this is the first study demonstrating a significant impact of sex on mucosal infection rates in rhesus macaques. One possibility is that the adjuvants alone differentially impacted infection risk in males versus females; discerning such effects requires studies comparing adjuvants in the absence of immunogen. Innate immune responses following vaccination also warrant investigation. Few studies have directly compared mucosal infection risk between sexes in either macaques or humans. In a previous report combining control animals from multiple challenge studies, intrarectal SIVmac251 infection rates appeared similar in male and female controls [41], although rates were not formally compared. Possible mechanisms underlying the difference observed here include: 1) higher type I interferon response to the SHIV challenge inoculum TLR7 stimulation in females limiting early viral replication [27], 2) greater propensity among males to produce TNF upon TLR4 stimulation (via the MPLA in ALFA) increasing viral transcription [43], and 3) estradiol-mediated inhibition of viral transcription in females via the repressive estrogen receptor in the LTR [44]. In humans, by contrast, a recent meta-analysis showed intrarectal HIV-1 acquisition risk to be higher in women than men [45], though only one study assessing intrarectal transmission risk in women was available. Taken together, there are limited and inconsistent findings regarding the impact of sex on mucosal transmission rates in humans and NHP and more studies involving females are needed. Establishing whether sex influences mucosal infection risk in other SHIV or SIV models will be important to ensure that future studies including both sexes are adequately powered to assess vaccine efficacy.

In summary, our findings strongly support future clinical and pre-clinical studies exploring liposomal adjuvants with MPLA and other next-generation immunostimulants aimed at developing a more effective HIV-1 vaccine. Other pathogens lacking protective vaccines may also benefit from incorporation of such adjuvants.

## Methods

### Study design, immunizations and SHIV challenge

Forty-eight colony-bred Indian-origin male and female 3-6-year-old rhesus macaques (*Macaca mulatta)* were assigned to three groups in a procedure resembling minimization. Group assignments were made deterministically by sorting all animals by *TRIM5* alleles, *TRIMcyp* positivity, pre-existing (cross-reactive) p27 ELISA titer, sex, weight, and age and then placing them into groups 1, 2, and 3 in order. Additional adjustments were made to further balance the groups across these factors. Animals expressing protective *Mamu* alleles A*01, B*08, and B*17 were excluded from the study. The sample size was chosen to achieve at least 80% power to detect 60% vaccine efficacy in an individual vaccine arm, using a two-sided log-rank test with 5% type I error. Animals were primed with 10^8^ pfu of either MVA lacking insert or an equal mixture of three recombinant live-attenuated MVA vaccines expressing HIV-1 *env* and *gag/pol* genes [23]. MVA vaccines (MVA-A/C/E) consisted of the: 1) MVA-CMDR, CM235 *env* and CM240 *gag/pol* (subtype CRF01_AE); 2) MVA-KEA, KNH1144 *env* and KER2008 *gag/pol* (subtype A); and 3) MVA-TZC, TZA125 *env* and TZA246 *gag/pol* (subtype C)[23]. The MVA inoculum was administered in 1.8 ml injections. Three boosting immunizations consisted of MVA and a multimeric subtype C gp145 protein (CO6980v0c22) administered contralaterally at 100 μg/dose in 0.5 ml at months 3, 6, and 12 following the prime [18]. All vaccinations were intramuscular, alternating right and left quadriceps for each product with each vaccination. gp145 was adsorbed to aluminum hydroxide (Alhydrogel, Brenntag) at 300 μg aluminum per 0.5 ml dose. ALF liposomes consisted of 1.145 mM phospholipids (DMPC:DMPG ratio = 9:1), 43% cholesterol, and 100 μg of synthetic monophosphoryl 3-deacyl lipid A (3D-PHAD, MPLA:phospholipid ratio = 1:8.8; Avanti Polar Lipids) per dose in PBS. For ALFA, the gp145-Alhydrogel suspension was used to reconstitute microfluidized and lyophilized small unilamellar ALF [46]. At month 15, animals were serially challenged intrarectally with an AID40 (1:750 dilution) of R5-tropic SHIV-1157ipd3N4 (Dr. Ruth Ruprecht, through the NIH AIDS Reagent Program, Division of AIDS, NIAID, NIH, #11689)[20] until viremic or for up to ten prespecified challenges. The challenge interval was increased from weekly to fortnightly following the third challenge to ensure accurate determination of the infecting challenge in animals with viremia onset greater than seven days post-challenge. Following the tenth challenge, the remaining uninfected animals were challenged with a five-fold greater dose for up to three additional challenges, after which one animal remained uninfected. The intrarectal animal infectious dose that results in 40% infection (AID40) for this stock was determined by titration on 15 naïve rhesus macaques as previously described [47]. All animals were vaccinated and challenged concurrently (24 animals each day, split over two days, half of each study arm), with both sexes distributed throughout. Animal handling was consistent throughout the study and no animal illnesses were noted. Weekly weight and temperature monitoring did not reveal variations following vaccination by study arm or animal sex.

### Ethical statement

All *in vivo* procedures were carried out in accordance to institutional, local, state, and national guidelines and laws governing research in animals including the Animal Welfare Act. Animal protocols and procedures were reviewed and approved by the Animal Care and Use Committee of both the US Army Medical Research and Material Command (USAMRMC, protocol 11355007.03) Animal Care and Use Review Office as well as the Institutional Animal Care and Use Committee of Bioqual, Inc. (protocol number 14-B077), where non-human primates were housed for the duration of the study. Bioqual, Inc. and the USAMRMC are both accredited by the Association for Assessment and Accreditation of Laboratory Animal Care and are in full compliance with the Animal Welfare Act and Public Health Service Policy on Humane Care and Use of Laboratory Animals.

### Binding antibody responses

Sera were assayed in triplicate for the presence of protein- or peptide-specific IgG antibodies by ELISA as previously described [32] except that horseradish peroxidase-labeled affinity-purified goat anti-monkey IgG (Kirkegaard & Perry Labs #5220–0333, polyclonal, Lot 10249162) was used as the secondary antibody. Briefly, 96-well Immulon 2 “U” bottom microtiter plate (Thermo Scientific) were coated with 0.1 μg/well of gp145 or gp70V1V2 protein for 2 hours at 37°C and then blocked overnight at 4°C with 0.5% casein and 0.5% BSA in phosphate-buffered saline (PBS) at 4°C, washed, and incubated for 1 h at 25°C with serial dilutions of test serum. Plates were washed, incubated with goat anti-monkey IgG (described above) for 1 h at 25°C, and washed again. ABTS substrate (KPL) was added for 1 h at 25°C. Plates were analyzed at 405 nm on a SpectraMax 250 plate reader (Molecular Devices). Matched pre-immune sera were used as the negative control for each animal. Data are expressed as endpoint titers defined as the highest dilution that yielded an optical density reading greater than or equal to twice that of the background values. Titers were calculated after subtracting the mean absorbance of triplicate wells lacking antigen. Trimeric subtype C CN54 gp140 protein is derived from HIV-1_97CN54_ strain (GenBank accession number AF286226). For the peptide ELISA, wells were coated overnight with 0.2 μg/well of Streptavidin followed by the addition of 0.1 μg/well of biotinylated cyclic-V2 or -V3 peptides (JPT Peptide Technologies) in bicarbonate buffer, pH 9.6, for 1 h at 37°C. The wells were blocked overnight with 0.5% milk, 0.1% Tween 20 in phosphate-buffered saline (PBS) pH 7.4 at 4°C. The rest of the procedure was similar to the protein ELISA described above.

Binding antibody multiplex assay (BAMA) was performed blinded to vaccine arm as previously described [48]. Linear epitope-specific binding rectal IgG responses were assessed blinded to vaccine arm by peptide array as previously described [49], with modifications for mucosal IgG analysis. Mucosal sample specific binding activity (SA) to each peptide was calculated as follows: fluorescence intensity / total IgG concentration in the mucosal sample (μg/ml) * dilution. Response magnitude to each epitope is defined as the highest binding signal to an individual peptide within the region referenced by the epitope name (e.g. “p58-60” indicates the range of peptides covered by the epitopes in the array library). Vaccine-induced binding response magnitude, or Binding Response, is calculated as the log2 fold difference between pre- and post-immunization, where fold difference = (post-immunization SA) / (the greater of the following: a. matched pre-immune SA; or b. non-specific SA); wherein non-specific SA is defined as: (median intensity of all pre-immune samples) / (IgG concentration of the post-immunization sample) * dilution. Non-specific SA represents the level of non-specific binding SA expected of a sample with the same IgG concentration as the post-immunization sample. Only 13 of 16 control animals were included in the array mapping assays for reference purposes; the three excluded animals were deemed “non-responders” by other binding antibody assays.

### Rapid Fluorometric Antibody-dependent Cellular Cytotoxicity (RFADCC) assay

RFADCC responses were measured pre and post-vaccination as described previously [50] using CEM.NKR CCR5+ target cells (NIH AIDS Reagent Program, #4376, by Dr. Alexandra Trkola)[51] pulsed with 30 μg/ml of the multimeric CO6980v0c22 gp145 protein for 1 hour. Cells were washed with ice-cold PBS, labeled with PKH26 (Sigma-Aldrich, St. Louis, MO) and then loaded with CFSE (Life Technologies Corp., Carlsbad, CA). Serum was added at 1:2500 dilution for opsonization at room temperature for 15 min. 50,000 HIV-negative human PBMCs were added at an effector to target ratio of 10:1 and incubated at 37°C for 4 hours. Washed, paraformaldehyde-fixed cells were acquired on a LSRII flow cytometer (Becton Dickenson, San Jose, CA) and analyzed using Flow Jo version 10.0.8 (TreeStar, Inc. Ashland, OR). The proportion of target cells that lost CFSE was quantified (S8E Fig). Percent lysis was determined using the formula: (CFSE^-^ PKH26^+^ / [CFSE^-^ PKH26^+^ + CFSE^+^ PKH26^+^]) *100. Unpulsed target cells treated similarly had a background lysis consistently below 5%.

### Natural killer cell intracellular cytokine staining

CO6980v0c22 gp145 protein (1.5 μg/ml) was coated onto 96-well plates overnight at 4°C and then washed with PBS. Purified IgG (35 μl of total Ig at 0.5 mg/ml) from serum was added, incubated at 25°C for 2 hours, and washed with PBS. Cryopreserved PBMCs from a single healthy human donor were added at 1 million cells/well and incubated at 37°C for 6 hours in presence of Brefeldin A and Monensin (BD Bioscience). Cells were stained with a cocktail of fluorescent-labeled antibodies (BD Biosciences unless otherwise indicated) for CD3-Alexa Fluor 700 (UCHT1, #557943, Lot 5061704), CD14-PE-Cy5 (TuK4, Invitrogen #MHCD1406, Lot 693377D), CD16-APC-Cy7 (3G8, #557758, Lot 5023818), CD19-PE-Cy5 (SJ25-C1, Invitrogen #MHCD1906, Lot 985754J), and CD56-PE-Cy7 (NCAM16.2, #335809, Lot 6050971) and with Live/dead Fixable Aqua Cell Stain (ThermoFisher). Cells were fixed and permeabilized (Cell Fixation & Permeabilization Kit, ThermoFisher) and stained intracellularly for TNFα-FITC (Mab11, BioLegend #502906, Lot B161818), IFNγ-BD Horizon V450 (B27, #560371, Lot 5275825), and MIP1β-PE (D21.1351, #550078, Lot 4189575). Data were acquired on a BD LSRII instrument and analyzed using FlowJo Version 9.9.6 software. NK cells were gated as follows: time gate to ensure steady flow stream, singlets (FSC-H v. FSC-A); aqua-negative; low side scatter; triple negative for CD3, CD19, and CD14; and CD56- and/or CD16-positive (S8F Fig).

### Antibody-dependent phagocytosis

ADCP was measured as previously described [15], using CO6980v0c22 gp145 (NHP study) or gp120 SF162 (Immune Technology; AVEG015 clinical study). Antigen was biotinylated following manufacturer’s instructions (ThermoFisher Scientific) and incubated with yellow-green streptavidin-fluorescent beads (Molecular Probes) overnight at 4°C. A 100-fold dilution of antigen-coated beads was incubated 2h at 37°C with diluted serum or plasma samples and washed before addition of THP-1 cells (Sigma Millipore, #88081201; 20,000 cells per well in duplicate). Following 19h incubation at 37°C, the cells were fixed with 2% formaldehyde solution (Tousimis) and fluorescence was evaluated on a LSRII (BD Biosciences). The phagocytic score was calculated by multiplying the percentage of bead-positive cells by the geometric mean fluorescence intensity of the bead-positive cells and dividing by 10^4^ (S8G Fig). Background responses assessed in the absence of serum or plasma were subtracted from all values.

For ADNP, CO6980v0c22 gp145 or gp120 SF162 was biotinylated following manufacturer’s instructions (ThermoFisher) and incubated with yellow-green streptavidin-fluorescent beads (Molecular Probes) for 2 hours at 37°C. Fresh peripheral blood leukocytes were used as effector cells and prepared from human or rhesus macaque blood by red blood cell lysis with ACK lysing buffer (ThermoFisher). Following HIV Env antigen biotinylation, 10μl of a 100-fold dilution of the bead–protein conjugate was incubated for 2h at 37°C with 100μl of 125-fold diluted serum or plasma and washed before addition of effector cells (50,000 cells/well). Fresh peripheral blood leukocytes were used as effector cells and prepared from human or rhesus macaque blood by red blood cell lysis with ACK lysing buffer (ThermoFisher). After 1h incubation at 37°C, cells were washed, surface stained, fixed with 2% formaldehyde solution, and evaluated on a LSRII (BD Biosciences). Antibodies used for flow cytometry were as follows for human leukocytes: CD3-Alexa Fluor 700 (UCHT1, BD Biosciences #557943, Lot 9050801), CD14-APC-Cy7 (MϕP9, BD Biosciences #557831, Lot 8184721), and CD66b-Pacific Blue (G10F5, BioLegend #305112, Lot B256448); and for rhesus leukocytes: CD3-Alexa Fluor 700 (SP34-2, BD Biosciences #557917, Lot 7146565), CD14-APC (M5E2, BD Biosciences #555399, Lot 7116537), and CD66abce-PE (TET2, Miltenyi Biotec #130-117-699, Lot 5180924891). The phagocytic score was calculated by multiplying the percentage of bead-positive neutrophils (SSC high, CD3- CD14- CD66+; S8H Fig) by the geometric mean fluorescence intensity of the bead-positive cells and dividing by 10^4^.

### Antigen-specific T cell responses

Cryopreserved PBMC were thawed, rested overnight in R10 with 50U/ml Benzonase Nuclease (Sigma-Aldrich), and stimulated with peptide pools for 6 h; bronchoalveolar lavage mononuclear cells were stimulated directly *ex vivo* for 16 h. Stimulations consisted of either HIV-1 Env potential T cell epitope pools (1μg/ml, NIH AIDS Reagent Program, 11551) or SIVmac239 Gag peptide pool (2 μg/ml, NIH AIDS Reagent Program, #12364) and were performed in the presence of Brefeldin A (0.7 μl/ml, GolgiPlug^™^, BD Cytofix/Cytoperm Kit, Cat. 555028). Env PTE peptide pools 1–3 (amino acids 4–585) were chosen as they cover the entire extracellular portion (gp120), plus 60 amino acids of the extracellular portion of gp41 (pool 3). Following stimulation, cells were stained serially with LIVE/DEAD Fixable Aqua (ThermoFisher #L34957) and a cocktail of fluorescent-labeled antibodies (BD Biosciences unless otherwise indicated) to cell surface markers CD4-BV605 (OKT4, BioLegend #317438, Lot B216636), CD8-BV711 (RPA-T8, BioLegend #301044, Lot B208239), CD45RA-PE.Cy5.5 (MEM-56, ThermoFisher #MHCD45RA18, Lot 1741577A), CD28-PE.Cy5 (CD28.2, #555730, Lot 6224858), CCR7-PE-CF594 (150503, #562381, Lot 6077673), CD95-BV421 (DX2, BioLegend #305624, Lot B224425). Intracellular cytokine staining was performed following fixation and permeabilization (BD Cytofix/Cytoperm, Becton Dickenson) with CD3-Cy7APC (SP34-2, #557757, Lot 6140803), CD154-Cy7PE (24–31, BioLegend #310832, Lot B203700), IFNγ-APC (4S.B3, #551385, Lot 6287841), TNFα-FITC (MAb11, #552889, Lot 6267512), and IL-2-BV650 (MQ1-17H12, BioLegend #500334, Lot B214940). Sample staining was measured on an LSR II (Becton Dickenson) and data analyzed using FlowJo v.9.9 software (Tree Star, Inc.). CD4 and CD8 T cell subsets were pre-gated on memory markers prior to assessing cytokine expression as previously described as follows: CD95+ and CD28+/- for CD4 T cells; single positive or double negative for CD45RA and CCR7 for CD8 T cells (S9 Fig). Boolean combinations of cells expressing one or more cytokines were used to calculate the sum total of antigen-specific memory CD4 or CD8 T cells. Statistical analysis and display of multicomponent distributions was performed with SPICE v5.3 (NIAID, NIH)[52]. Response functionality and polyfunctionality was also determined by COMPASS [8].

### Primary immune correlates analysis

The goal of the primary analysis was to determine potential immune correlates of protection. First, immunogenicity data from all assays were analyzed as follows. Assay values below the lower limit of detection (LOD) were imputed as LOD/2. For Fc array data, the mean of the replicates was used for all analyses; intraclass correlation coefficients were estimated for all probes to determine whether variability between replicates was a concern and a sensitivity analysis using one randomly chosen replicate was needed. Titer data were log-2 transformed, and all variables were mean-centered and scaled by the sample standard deviation and treated as continuous for the primary analysis. Assays with little to no variability were excluded from consideration for analysis. For all analyses, *P*-values were adjusted to control the false discovery rate via the Benjamini-Hochberg procedure [53], within related subsets of assays (e.g. non-neutralizing effector antibody, Fc array, IgG titers), such that FDR is controlled within a class of assays. Tests with raw *P*-values less than 0.05 and adjusted p-values less than 0.2 were considered significant. Assays at 12.5 and 15 months were analyzed separately, and the *P*-value adjustment was done within each set of hypothesis tests (i.e. HRs in males, in females, and all animals separately).

To reduce the number of assays assessed in the primary analysis, the correlation between assays was examined to determine assays that appear redundant due to similar patterns with other assays (S1 Table). Spearman rank-order correlation coefficients were estimated between all pairs of assays within a class (e.g. IgG titers), and the assay highly correlated (rho>0.7) with the greatest number of other assays was removed until the correlation between all remaining pairs fell below this threshold. If two or more assays were highly correlated with the same number of other assays, one was randomly chosen to remove at that step. For completeness, all assays were analyzed in an exploratory analysis (S3 Table).

The assays remaining after the filtering steps were individually assessed for associations with the risk of infection via Cox proportional hazards models (S2 Table). Sex and vaccine arm were included as covariates, along with a sex by assay interaction term to allow for differing patterns of protection between sexes. Similar models were fit without the interaction terms to examine combined-sex associations. Baseline values were also included where available, to adjust for the background noise and possible innate immune responses. Control animals were included in the primary analysis for assays performed for all animals, but they were dropped from sensitivity analyses to assess vaccine-induced effects.

### Phase 1 clinical trial study design

AVEG015 was a phase 1 randomized double-blind, placebo-controlled, clinical trial conducted in 1992 in the United States, sponsored by the National Institute of Allergy and Infectious Diseases, National Institutes of Health (Bethesda, MD), to compare the safety and immunogenicity of CHO-expressed HIV-1_SF-2_ rgp120 (Biocine), individually combined with 7 adjuvants in a total of 112 HIV-1-uninfected low-risk individuals (18–60 years)[32]. Archived sera from the aluminum hydroxide arm (HIV-1_SF-2_ gp120 adsorbed to Alhydrogel) and the liposomal arm (L[HIV-1_SF-2_ gp120 + MPL] adsorbed to Alhydrogel) were used (n = 9–13 participants/arm per time point for available specimens; n = 6 females per arm, n = 7 males per arm). Vaccinations occurred at weeks 0, 8, 24 and 72 with 50 μg of gp120 and 0.5 mg of aluminum (aluminum hydroxide arm) or 37.5 μg of encapsulated gp120, 2.2 mg of MPL (RIBI Immunochem Research, Inc.), and 0.5 mg of aluminum (liposomal arm). The liposomes consisted of dimyristoyl phosphatidylcholine, dimyristoyl phosphatidylglycerol, cholesterol, and MPL [33]. These liposomes contained identical lipids as ALFA, but differed in the formulation as follows: 1) gp120 was encapsulated in the liposomes with extensive washing to remove free antigen, prior to liposome adsorption to aluminum; 2) liposomes were multilamellar rather than unilamellar; 3) the phospholipid:lipid A ratio was greater; and 4) the MPLA content was greater. Samples were obtained under an HIV Vaccine Trials Network ancillary study as a limited data set with no identifiers. *In vitro* assays were carried out under the Walter Reed Army Institute of Research Institutional Review Board approval (no.1741/RV313). All individuals provided informed consent to participate in the parental study.

### Statistical analysis

Animals that were not infected in the first 10 challenges were considered to be censored while still uninfected after the 10th challenge. For the purposes of the survival analysis, the discrete infecting challenge (i.e. 1, 2, …, 10) was treated as the failure time. Due to the relatively large proportion of uninfected animals left after the tenth challenge, the heterogeneous vaccine effect model from Nolen *et al*. was adapted to estimate overall vaccine efficacy and compare infection incidence between study arms, via likelihood ratio tests [54]. A Cox proportional hazards model with an interaction term between study arm and sex was used to assess sex-specific differences in vaccine efficacy.

## Supporting information

S1 Fig Lamina propria inflammatory markers and rectal microbiome. **(A)** Type 1 IFN responses (Mx1) and gastrointestinal tract dysfunction (neutrophil infiltration; MPO) in the lamina propria was quantitated one month pre-challenge by immunohistochemistry. Results are shown by animal sex for all animals (left) and by vaccine arm (right). **(B)** Microbial populations in the rectal mucosa were sequenced in all animals at study month 12. The relative abundance of the indicated microbial Families was compared between male and female macaques using unpaired t-test; *P* value is indicated. Error bars depict mean and standard deviation.
(TIF)

S2 Fig SHIV plasma viral load and sequencing. **(A)** Longitudinal viremia following intrarectal SHIV-1157ipd3N4 infection is shown for each animal by study arm, synchronized by time of infection. Cross indicates mortality. **(B)** Viral load peak, set point, and area under the curve (AUC, through week 24 PI) are compared across the study groups. Set point viral load was calculated as the average viral load of all samples collected between day 49 PI and until the end of the study (day 175 PI); n.s., not significant. Box plots depict median and interquartile range; whiskers reflect +/- 1.5*(interquartile range). **(C)** Comparison of set point viral load between sexes within each study arm is shown as violin plots. Median values and significant *P* values (Wilcoxon rank-sum test) are indicated. **(D)** Highlighter plot depicts single genome sequencing of 33 *env* sequences from the SHIV-1157ipd3N4 challenge viral stock (top) and 10 plasma *env* sequences from each of two female animals in the control arm, 6310 and 6133, four weeks post-infection (bottom).
(TIF)

S3 Fig Env-specific serum binding antibody responses to additional Env antigens. Env-specific IgG responses to HIV-1 antigens in serum two weeks following the fourth immunization (month 12.5) and at the time of challenge (month 15) were measured by ELISA **(A)** and BAMA (**B-D**). For BAMA, the area under the curve (AUC) is reported. Responses are shown for the indicated strains for gp140, gp120, gp41 and gp70-scaffolded V1V2 antigens. **(E)** CD4 binding site (bs) and CD4-induced (CD4i) Env-specific responses are depicted as the MFI ratio between wild-type Env and the indicated mutation. Ratios are calculated only when binding to WT meets positivity criteria (see “Methods” for positivity criteria) and MFI for mutant >50. **(F)** Infected cell binding antibodies (ICABA) were assessed as the percentage of SHIV-1157 infected cells (p24+) stained by animal serum (left) and the median fluorescence intensity (MFI, right) of the infected cell staining. Gray bars reflect the interquartile range; black lines depict medians; *P* values reflect Wilcoxon rank-sum test.
(TIF)

S4 Fig Clustering of vaccine arms by binding antibody responses assessed by Fc array. **(A)** Hierarchical clustering of treatment arms by biophysical properties at peak immunity (month 12.5, left) and first challenge (month 15, right). Each row represents a full binding antibody profile for a single animal with each cell representing a z-score of the response. Columns represent clustered Env-specific antibody responses, generalized by the antibody detection reagent (top row) and Env protein antigen (bottom row) shown by the colored bars along the top. Composite scores were generated for each Env antigen (gp140, gp120, V1V2, and V3) for clustering analysis. **(B)** Principal component analysis (PCA) of binding antibody responses assessed by Fc array among vaccinated animals (no controls) at peak and first challenge. No separate or distinct profiles specific to the ALFA or alum arms were observed. The major variation captured along the first principal (46.2% at 12.5 months and 61.7% at 15 months) was due to vaccine-elicited immune responses, but not a specific adjuvant (alum or ALFA).
(TIF)

S5 Fig Env-specific rectal mucosa binding antibody responses to multiple Env antigens. Env-specific IgG and IgA responses to HIV-1 antigens in rectal mucosa two weeks following the fourth immunization (month 12.5) were measured by BAMA. (**A-C**) IgG responses are shown for the indicated strains for gp140 antigens **(A)**, subtype C unless otherwise indicated; gp120 and gp41 antigens **(B)**; and gp70-scaffolded V1V2 **(C)**. **(D)** IgA responses are shown for the immunogen, challenge strain antigens, and Con S gp140. Specific activity (SA) was calculated as the MFI * dilution / IgG or IgA concentration. Gray bars reflect the interquartile range; black lines depict medians; *P* values reflect Wilcoxon rank-sum test differences between IgG responses in active arms and IgA responses among any arms.
(TIF)

S6 Fig Env-specific rectal mucosal binding antibody responses mapped by linear peptide array. Rectal Env-specific IgG responses to linear peptides from multiple HIV-1 strains were measured by peptide microarray at peak immunogenicity (month 12.5). **(A)** Heat maps depict vaccine group median binding response magnitude (top, log_2_ fold difference (Fd) post-/pre-immunization) and positivity rate (bottom) by HIV-1 Env strain (rows). The peptide library used for mapping includes 15-mer peptides overlapping by 12. Linear peptide numbers based on sequence alignment are indicated at bottom for gp120 (left) and gp41 (right). Gray shading in the heat map indicates peptide missing in the peptide alignment at the indicated peptide number (due to a deletion of more than 2aa in sequence). Gp120 constant and variable regions as well as notable gp41 regions are indicated along the top. **(B)** Scatterplots of the individual animal epitope-specific binding responses by vaccine arm for four consensus Env strains. Percentage of animals positive within each active arm are indicated at top. Symbol and text color reflect control (black), alum (red), and ALFA (blue) groups. Box plots depict median, interquartile range, and +/- 1.5 the interquartile range. **(C)** Definition of the epitopes presented in scatterplots in (B).
(TIF)

S7 Fig Vaccine-elicited neutralizing antibody responses. **(A)** Longitudinal pseudovirus neutralization by vaccinated animal sera was measured in TZM-bl target cells at the indicated month post first vaccination. Neutralization sensitive tier 1 pseudoviruses GS015 (tier 1, subtype C), MW965 (tier 1, subtype C), SF162 (tier 1, subtype B), and TH023 (tier 1, subtype AE) were measured. The serum dilution that resulted in 50% inhibition (ID_50_) is plotted for each animal by vaccine group. No responses were detected against the SHIV challenge strain, five acute subtype C tier 2 pseudoviruses, or MuLV (negative control). Tested placebos were negative in all assays. Neutralization breadth **(B)** and potency **(C)** scores were determined for each animal from the active arms and shown at peak and time of challenge. Gray bars reflect the interquartile range; black lines depict medians; *P* values reflect Wilcoxon rank-sum test differences between active arms.
(TIF)

S8 Fig Antibody effector responses stratified by sex, assessed with rhesus neutrophil effectors, and gating methodology. **(A)** gp145 Env-specific ADCP and ADNP **(B)** responses stratified by sex. **(C)** gp145-specific ADNP responses measured using fresh rhesus PBMC neutrophils. Serum was used at month 0 and plasma at months 12.7 and 15 due to sample availability. Open symbol depicts animal that resisted the ten prespecified and three supplemental high-dose challenges. Gray boxes depict interquartile range; black bars are medians; Wilcoxon rank-sum test *P* values are shown. **(D)** Correlation between ADNP phagocytic scores obtained using either rhesus (y-axis) or human (x-axis) neutrophils is shown at peak immunogenicity (top, month 12.7) and time of challenge (month 15) with and without the outlier animal (bottom). Spearman’s rho (ρ) and p-value are indicated. **(E)** Flow cytometry gating scheme used to quantitate ADCC is shown for representative serum samples collected at baseline and at month 12.5 (animal 6303). Sequential gating (left two plots) was used to identify PKH26-labeled CEM target cells, followed by the fraction lysed target cells (CFSE-negative, right). **(F)** Flow cytometry gating scheme used to quantitate NK ICS is shown for representative serum samples collected at baseline and at month 12.5 (animal 6324). Sequential gating (top row, left to right, followed by CD56 and CD16 gating, middle and bottom rows) was used to identify NK cells. All cytokine gates were drawn on the NK population depicted at left. **(G)** Flow cytometry gating scheme used to quantitate monocytic THP-1 ADCP is shown for representative serum and plasma samples collected at baseline (left) and month 12.7 (right) from a vaccinated animal (6127). **(H)** Flow cytometry gating scheme used to quantitate ADNP using human leukocytes is shown for representative serum and plasma samples. Sequential gating (left to right) was used to identify neutrophils, followed by histogram gating of bead-positive neutrophils. Baseline (left) and month 12.7 (right) histograms depict a vaccinated animal (6129).
(TIF)

S9 Fig Antigen-specific CD4 T cell response quality and flow cytometry gating. Vaccine-elicited PBMC HIV-1 Env-specific CD4 T cells were measured by ICS for IFNγ, TNFα, IL-2, and CD154 following *ex vivo* Env PTE peptide pool stimulation. (**A-B**) Response functionality was assessed by Boolean cytokine combinations represented within the HIV-specific response following the third immunization (month 6 + 3 wks) using SPICE. Bar graphs depict the fraction of the response comprised by each Boolean combination for each animal grouped by vaccine arm; pie charts show the average for each active arm. Predominant Boolean combinations are annotated in the pie charts for reference. (**C-D**) Longitudinal PBMC CD4 T cell functionality and polyfunctionality scores assessed by COMPASS through week 4 post-infection (PI). Gray bars depict interquartile range; black lines depict medians; No significant differences between active arms were observed. **(E)** Flow cytometry gating scheme used to identify and quantitate antigen-specific T cell in PBMC is shown from a representative sample (animal 6306, month 12.7, Env PTE pool 3). Sequential gating (top row, left to right) was used to identify T lymphocytes, followed by memory CD4 (middle row) and CD8 (bottom row) gating. All cytokine gates shown were performed with SSC-A on the x-axis.
(TIF)

S10 Fig Rectal mucosal IgG envelope peptide microarray responses associated with reduced risk of infection. HIV-1 envelope-specific IgG responses were mapped by peptide microarray for rectal secretions at peak immunogenicity (month 12.5). **(A)** Heatmap depicts association between peak rectal epitope-specific responses and infection risk. Hazard ratios for all peptide array epitope responses that remained in the analysis set after filtering out highly correlated responses are shown, separately by sex and in all animals (*, p<0.05). **(B)** Epitope-specific binding responses that were protective in males with p<0.2 are shown for each animal by vaccine arm. Outlier box plots depict median, interquartile range, and +/- 1.5 the interquartile range. Female animals are depicted by circles, male by triangles. Open symbols reflect animals that remained uninfected following the ten prespecified challenges.
(TIF)

S1 Table Correlations between immune responses within an assay class. Immunogenicity data from all humoral and cellular responses assessed was divided into assay classes and by time point (peak, month 12.5; time of challenge, month 15) when available, represented by tabs within the table. Correlations between assays within a class were determined by Spearman rank-order and highly correlated assays (rho>0.7) are indicated by pink shading and red text.
(ZIP)

S2 Table Immune correlates of protection primary analysis. Associations between immune responses and SHIV infection risk were assessed by Cox proportional hazards models for assays that remained after filtering out highly correlated assays. Results are grouped by related immunologic assays and time point, represented by different tabs within the spreadsheet. Sex and vaccine arm were included as covariates, along with a sex by assay interaction term to allow for differing patterns of protection between sexes. Assays with protective effects that met the significance threshold of unadjusted *P*<0.05 and FDR-adjusted q<0.2 are highlighted in blue, while assays with *P*<0.1 but q>0.2 are highlighted in light purple. Analyses that included active arms only (“vaccinees only”), were baseline-controlled, and adjusted for outliers are indicated. The proportional hazards (PH) assumption was tested for the sex-specific and combined-sex models; small p-values indicate that this assumption may be invalid, which would prompt additional caution in interpreting the results, particularly the p-values. Abbreviations: SD, standard deviation; HR, hazard ratio.
(XLSX)

S3 Table Immune correlates of protection analysis including all assays. Associations between immune responses and SHIV infection risk were assessed by Cox proportional hazards models for all assays (without filtering out highly correlated assays). Analysis was performed as described in S2 Table. Abbreviations: SD, standard deviation; HR, hazard ratio.
(XLSX)

S4 Table Immune correlates of protection analysis in active vaccine arms only. Associations between immune responses and SHIV infection risk were assessed by Cox proportional hazards models as in S2 Table but for all assays and restricted to vaccinated animals only (no controls). Abbreviations: SD, standard deviation; HR, hazard ratio; PH, proportional hazards.
(XLSX)

S5 Table Immune correlates of protection analysis in active vaccine arms only and without adjustment for study arm. Associations between immune responses and SHIV infection risk were assessed by Cox proportional hazards models as in S2 Table but for all assays, restricted to vaccinated animals, and without adjustment for study arm. Abbreviations: SD, standard deviation; HR, hazard ratio; PH, proportional hazards.
(XLSX)

S6 Table Multi-assay immune correlates of protection at peak immunogenicity. Associations between immune responses and SHIV infection risk were assessed by lasso-penalized Cox regression in all vaccinated animals with sex, vaccine arm, and all peak (month 12.5) immunogenicity assay data as covariates. The ten protective assays and study arm that remained in the model after all other coefficients were shrunken to zero are listed. Abbreviations: HR, hazard ratio; CI, confidence interval.
(XLSX)

S1 File Supplementary methods. (DOCX)
