# Supplementary figures and images for: Adjuvanted HIV-1 vaccine promotes antibody-dependent phagocytic responses and protects against heterologous SHIV challenge
Source: PLoS Pathog. 2020 Sep 3;16(9):e1008764. doi: 10.1371/journal.ppat.1008764 (PMC7505435; doi:10.1371/journal.ppat.1008764)

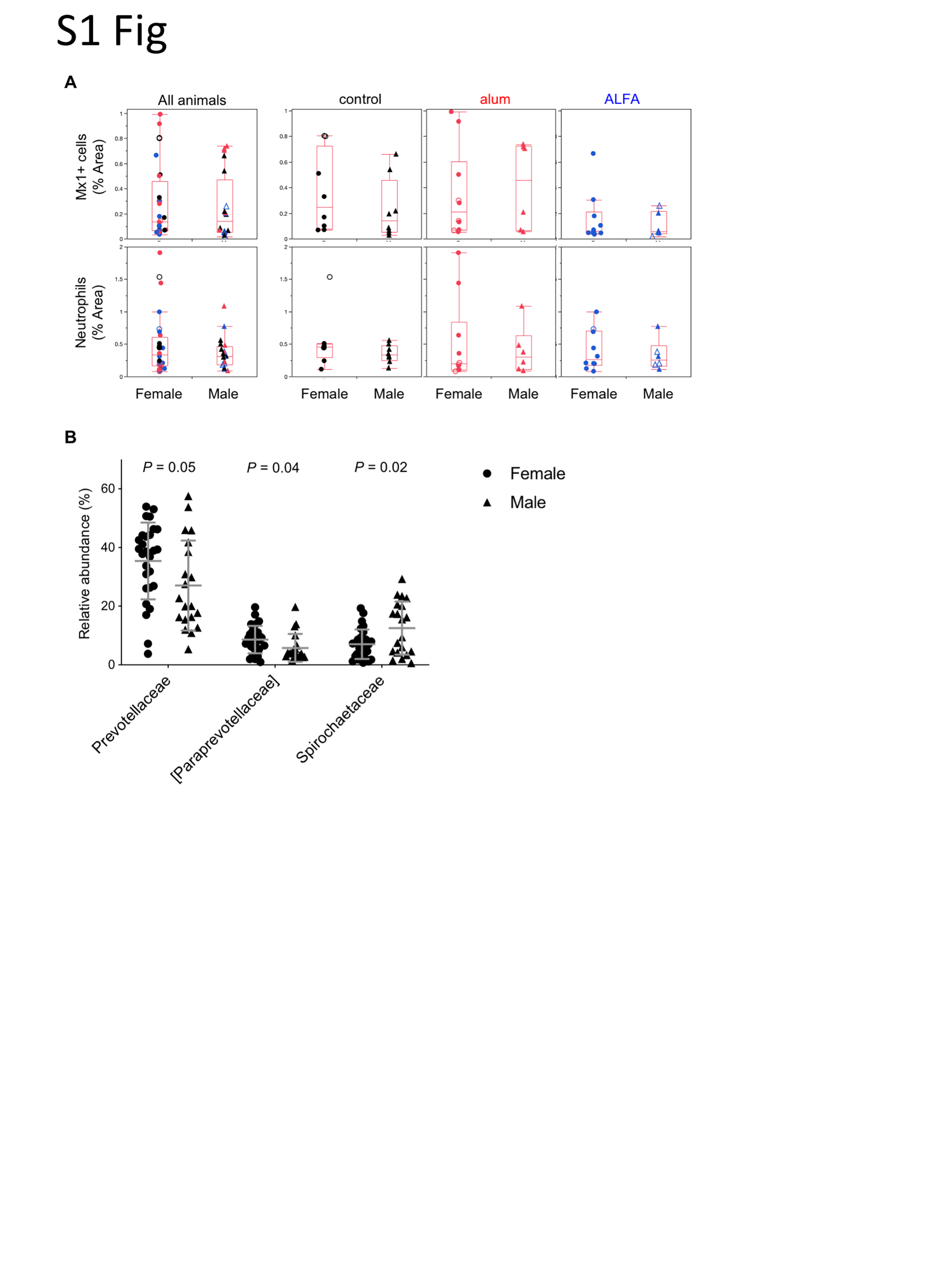

Supplement: S1 Fig — (A) Type 1 IFN responses (Mx1) and gastrointestinal tract dysfunction (neutrophil infiltration; MPO) in the lamina propria was quantitated one month pre-challenge by immunohistochemistry. Results are shown by animal sex for all animals (left) and by vaccine arm (right). (B) Microbial populations in the rectal mucosa were sequenced in all animals at study month 12. The relative abundance of the indicated microbial Families was compared between male and female macaques using unpaired t-test; P value is indicated. Error bars depict mean and standard deviation. (TIF) [file ppat.1008764.s001.tif]

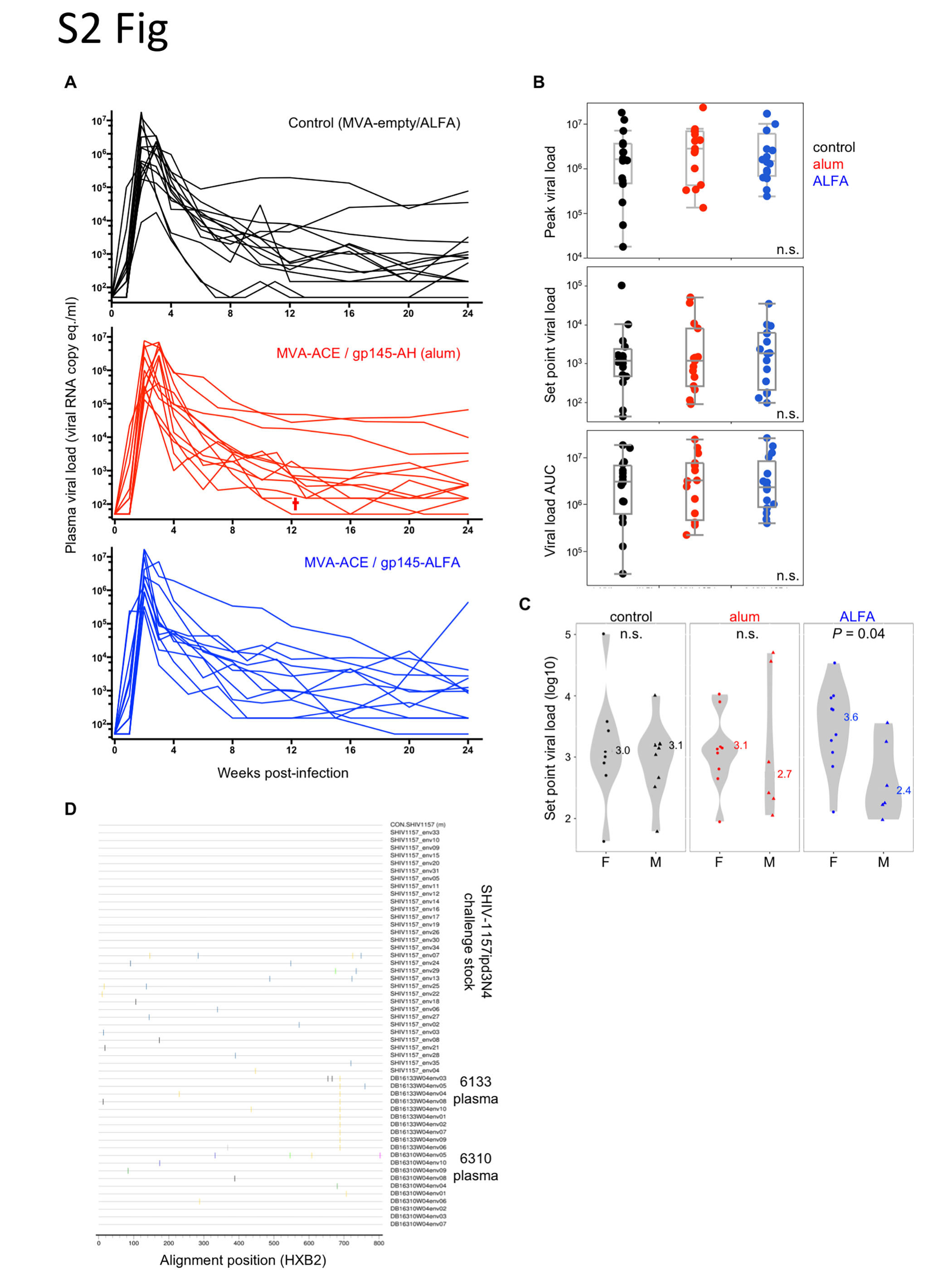

Supplement: S2 Fig — (A) Longitudinal viremia following intrarectal SHIV-1157ipd3N4 infection is shown for each animal by study arm, synchronized by time of infection. Cross indicates mortality. (B) Viral load peak, set point, and area under the curve (AUC, through week 24 PI) are compared across the study groups. Set point viral load was calculated as the average viral load of all samples collected between day 49 PI and until the end of the study (day 175 PI); n.s., not significant. Box plots depict median and interquartile range; whiskers reflect +/- 1.5*(interquartile range). (C) Comparison of set point viral load between sexes within each study arm is shown as violin plots. Median values and significant P values (Wilcoxon rank-sum test) are indicated. (D) Highlighter plot depicts single genome sequencing of 33 env sequences from the SHIV-1157ipd3N4 challenge viral stock (top) and 10 plasma env sequences from each of two female animals in the control arm, 6310 and 6133, four weeks post-infection (bottom). (TIF) [file ppat.1008764.s002.tif]

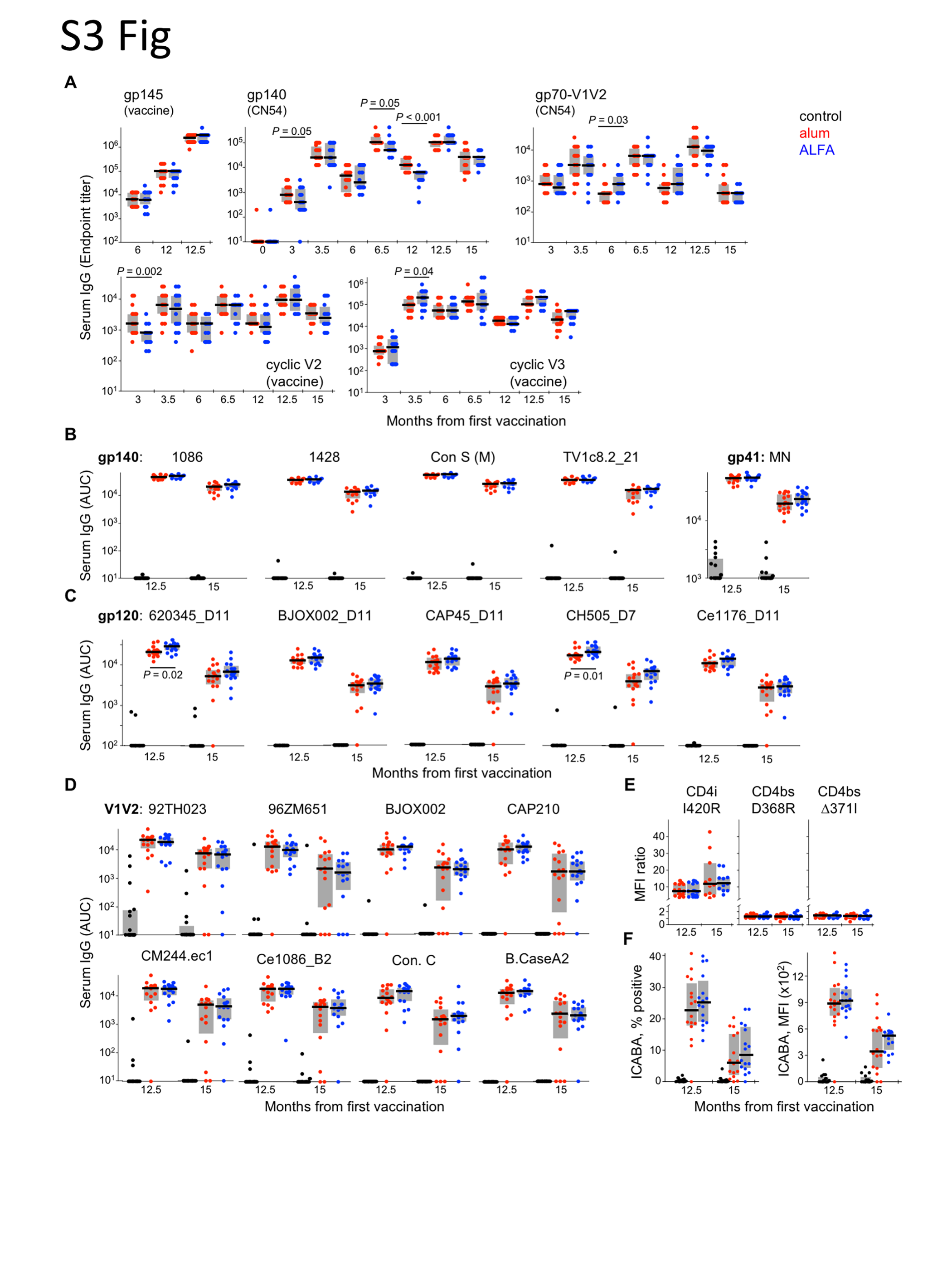

Supplement: S3 Fig — Env-specific IgG responses to HIV-1 antigens in serum two weeks following the fourth immunization (month 12.5) and at the time of challenge (month 15) were measured by ELISA (A) and BAMA (B-D). For BAMA, the area under the curve (AUC) is reported. Responses are shown for the indicated strains for gp140, gp120, gp41 and gp70-scaffolded V1V2 antigens. (E) CD4 binding site (bs) and CD4-induced (CD4i) Env-specific responses are depicted as the MFI ratio between wild-type Env and the indicated mutation. Ratios are calculated only when binding to WT meets positivity criteria (see “Methods” for positivity criteria) and MFI for mutant >50. (F) Infected cell binding antibodies (ICABA) were assessed as the percentage of SHIV-1157 infected cells (p24+) stained by animal serum (left) and the median fluorescence intensity (MFI, right) of the infected cell staining. Gray bars reflect the interquartile range; black lines depict medians; P values reflect Wilcoxon rank-sum test. (TIF) [file ppat.1008764.s003.tif]

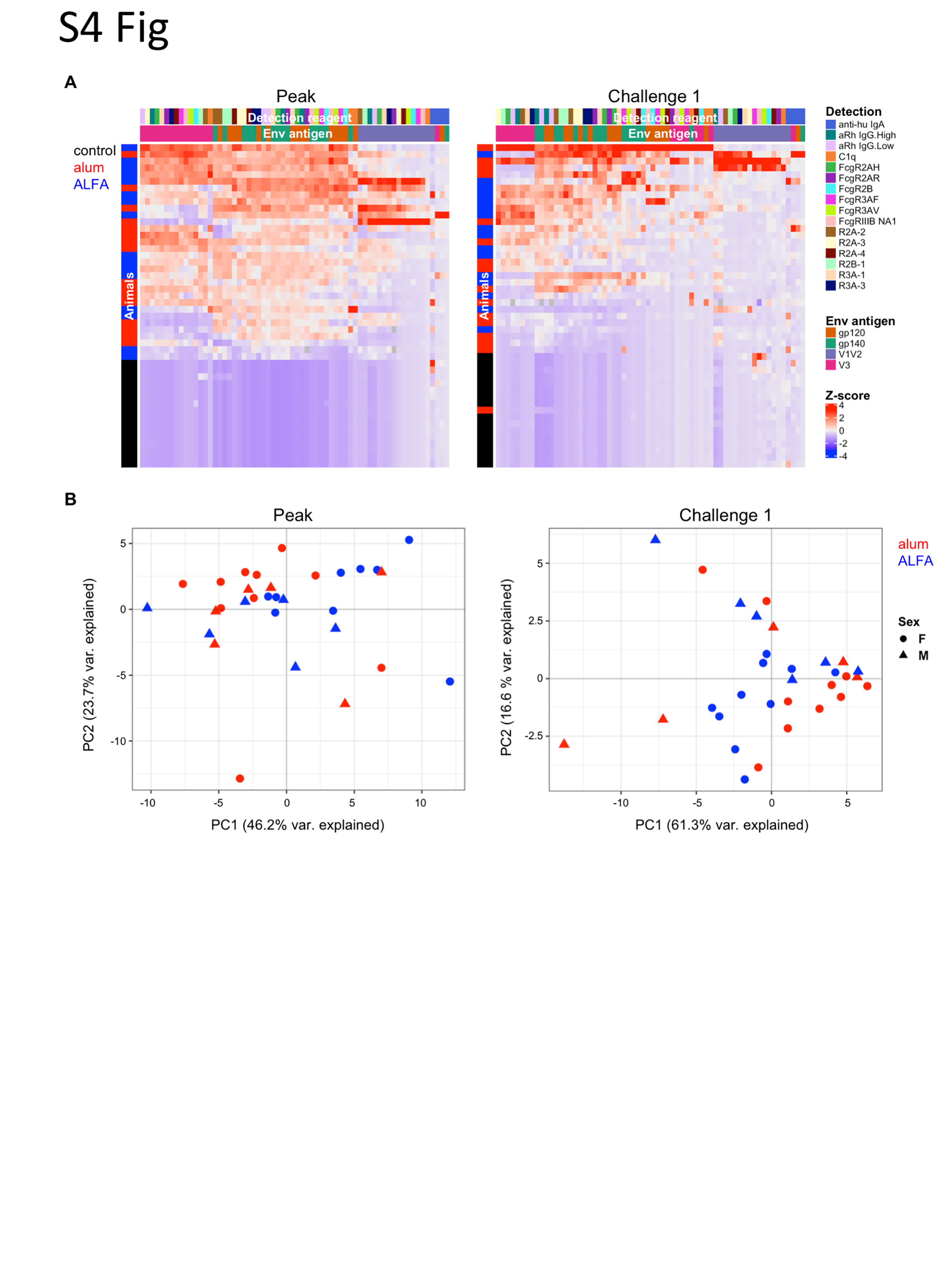

Supplement: S4 Fig — (A) Hierarchical clustering of treatment arms by biophysical properties at peak immunity (month 12.5, left) and first challenge (month 15, right). Each row represents a full binding antibody profile for a single animal with each cell representing a z-score of the response. Columns represent clustered Env-specific antibody responses, generalized by the antibody detection reagent (top row) and Env protein antigen (bottom row) shown by the colored bars along the top. Composite scores were generated for each Env antigen (gp140, gp120, V1V2, and V3) for clustering analysis. (B) Principal component analysis (PCA) of binding antibody responses assessed by Fc array among vaccinated animals (no controls) at peak and first challenge. No separate or distinct profiles specific to the ALFA or alum arms were observed. The major variation captured along the first principal (46.2% at 12.5 months and 61.7% at 15 months) was due to vaccine-elicited immune responses, but not a specific adjuvant (alum or ALFA). (TIF) [file ppat.1008764.s004.tif]

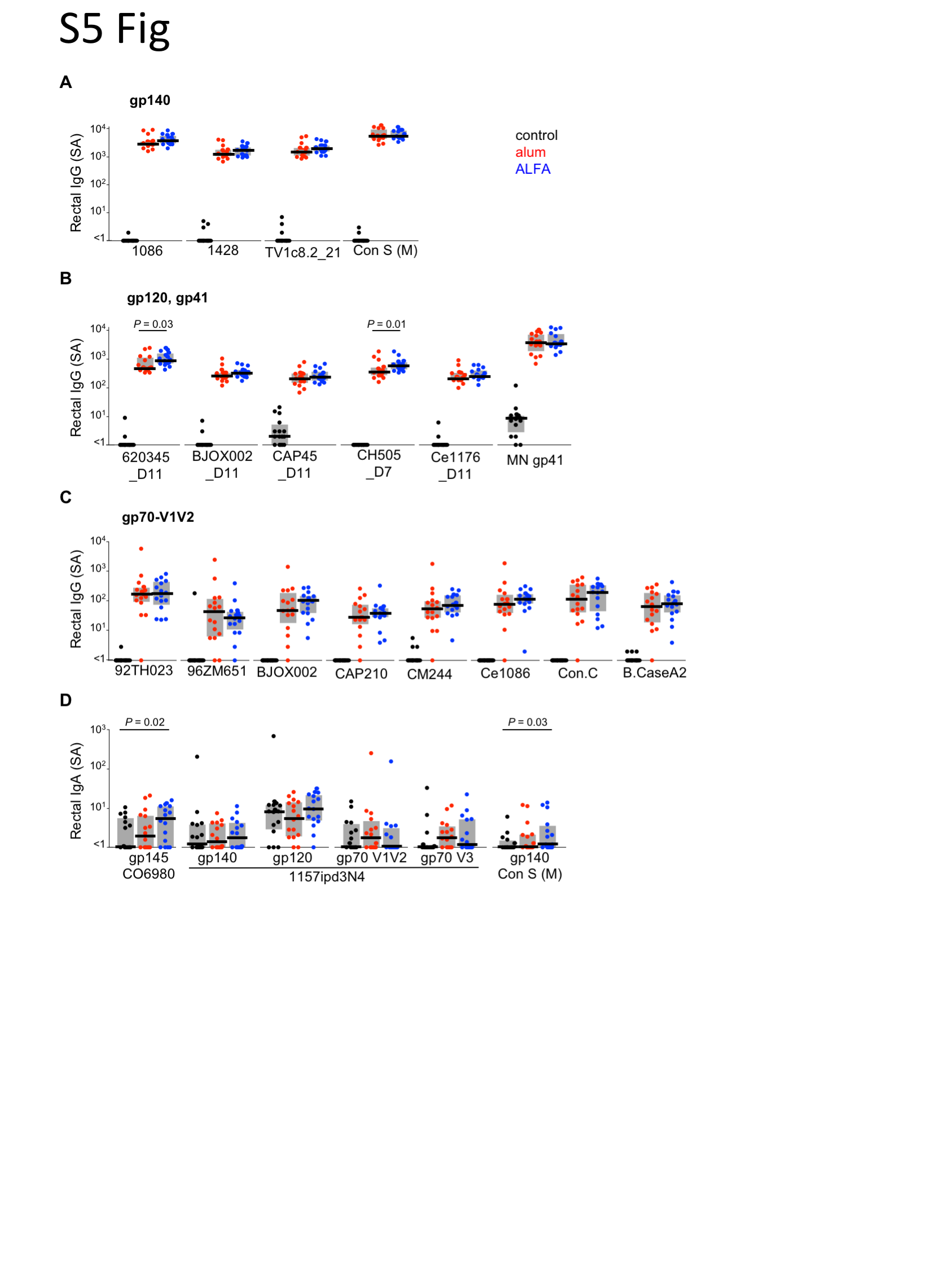

Supplement: S5 Fig — Env-specific IgG and IgA responses to HIV-1 antigens in rectal mucosa two weeks following the fourth immunization (month 12.5) were measured by BAMA. (A-C) IgG responses are shown for the indicated strains for gp140 antigens (A), subtype C unless otherwise indicated; gp120 and gp41 antigens (B); and gp70-scaffolded V1V2 (C). (D) IgA responses are shown for the immunogen, challenge strain antigens, and Con S gp140. Specific activity (SA) was calculated as the MFI * dilution / IgG or IgA concentration. Gray bars reflect the interquartile range; black lines depict medians; P values reflect Wilcoxon rank-sum test differences between IgG responses in active arms and IgA responses among any arms. (TIF) [file ppat.1008764.s005.tif]

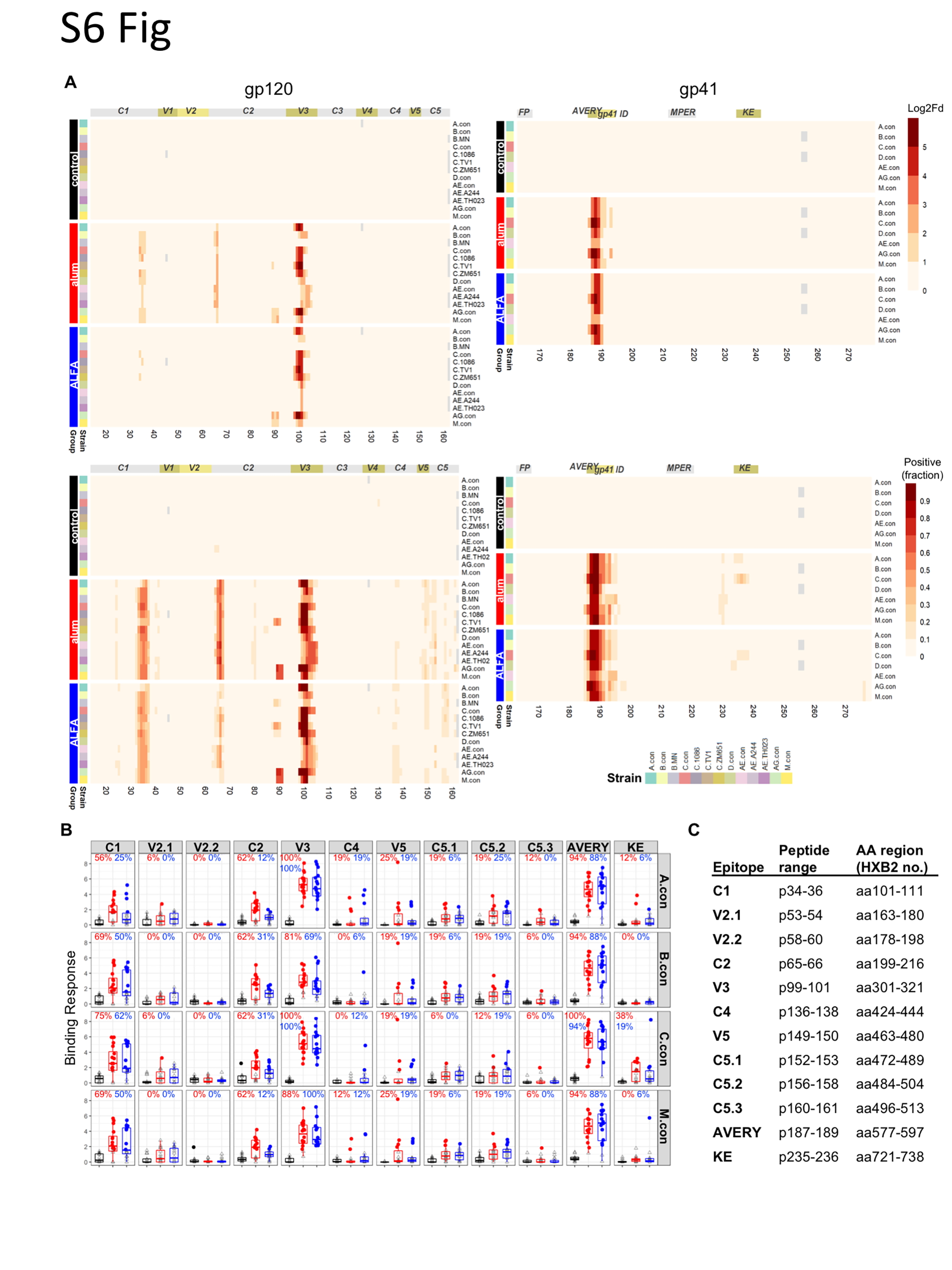

Supplement: S6 Fig — Rectal Env-specific IgG responses to linear peptides from multiple HIV-1 strains were measured by peptide microarray at peak immunogenicity (month 12.5). (A) Heat maps depict vaccine group median binding response magnitude (top, log2 fold difference (Fd) post-/pre-immunization) and positivity rate (bottom) by HIV-1 Env strain (rows). The peptide library used for mapping includes 15-mer peptides overlapping by 12. Linear peptide numbers based on sequence alignment are indicated at bottom for gp120 (left) and gp41 (right). Gray shading in the heat map indicates peptide missing in the peptide alignment at the indicated peptide number (due to a deletion of more than 2aa in sequence). Gp120 constant and variable regions as well as notable gp41 regions are indicated along the top. (B) Scatterplots of the individual animal epitope-specific binding responses by vaccine arm for four consensus Env strains. Percentage of animals positive within each active arm are indicated at top. Symbol and text color reflect control (black), alum (red), and ALFA (blue) groups. Box plots depict median, interquartile range, and +/- 1.5 the interquartile range. (C) Definition of the epitopes presented in scatterplots in (B). (TIF) [file ppat.1008764.s006.tif]

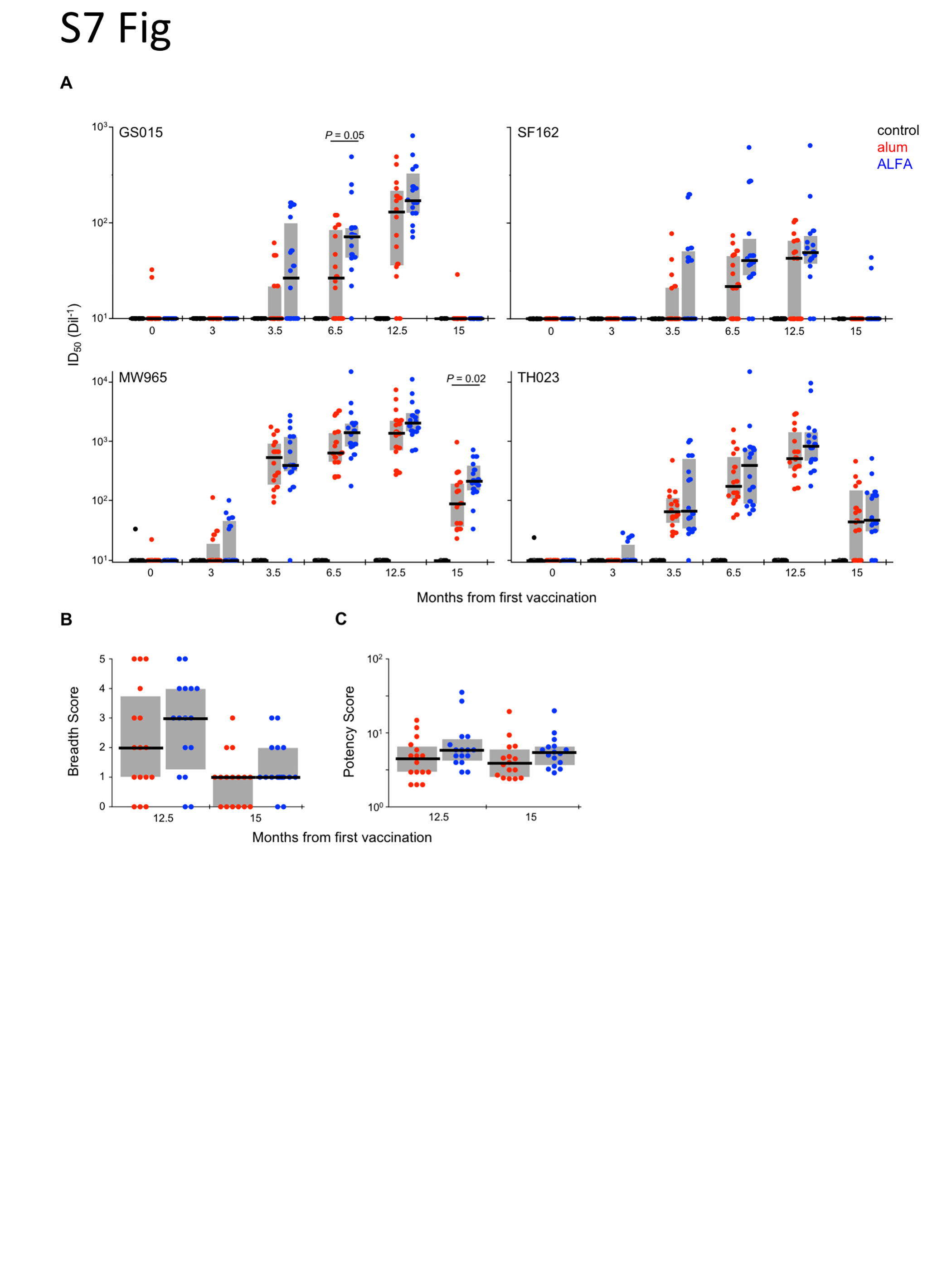

Supplement: S7 Fig — (A) Longitudinal pseudovirus neutralization by vaccinated animal sera was measured in TZM-bl target cells at the indicated month post first vaccination. Neutralization sensitive tier 1 pseudoviruses GS015 (tier 1, subtype C), MW965 (tier 1, subtype C), SF162 (tier 1, subtype B), and TH023 (tier 1, subtype AE) were measured. The serum dilution that resulted in 50% inhibition (ID50) is plotted for each animal by vaccine group. No responses were detected against the SHIV challenge strain, five acute subtype C tier 2 pseudoviruses, or MuLV (negative control). Tested placebos were negative in all assays. Neutralization breadth (B) and potency (C) scores were determined for each animal from the active arms and shown at peak and time of challenge. Gray bars reflect the interquartile range; black lines depict medians; P values reflect Wilcoxon rank-sum test differences between active arms. (TIF) [file ppat.1008764.s007.tif]

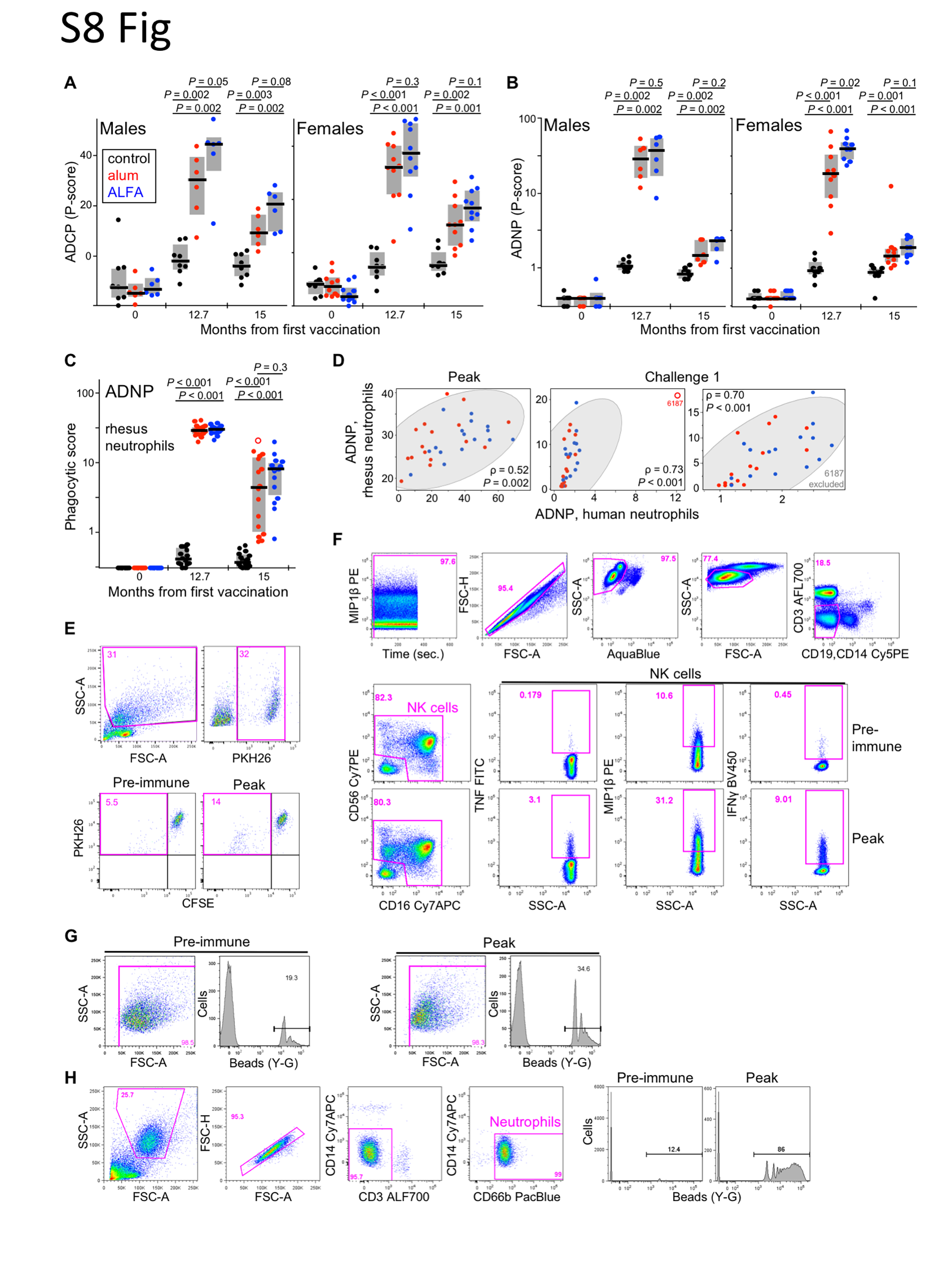

Supplement: S8 Fig — (A) gp145 Env-specific ADCP and ADNP (B) responses stratified by sex. (C) gp145-specific ADNP responses measured using fresh rhesus PBMC neutrophils. Serum was used at month 0 and plasma at months 12.7 and 15 due to sample availability. Open symbol depicts animal that resisted the ten prespecified and three supplemental high-dose challenges. Gray boxes depict interquartile range; black bars are medians; Wilcoxon rank-sum test P values are shown. (D) Correlation between ADNP phagocytic scores obtained using either rhesus (y-axis) or human (x-axis) neutrophils is shown at peak immunogenicity (top, month 12.7) and time of challenge (month 15) with and without the outlier animal (bottom). Spearman’s rho (ρ) and p-value are indicated. (E) Flow cytometry gating scheme used to quantitate ADCC is shown for representative serum samples collected at baseline and at month 12.5 (animal 6303). Sequential gating (left two plots) was used to identify PKH26-labeled CEM target cells, followed by the fraction lysed target cells (CFSE-negative, right). (F) Flow cytometry gating scheme used to quantitate NK ICS is shown for representative serum samples collected at baseline and at month 12.5 (animal 6324). Sequential gating (top row, left to right, followed by CD56 and CD16 gating, middle and bottom rows) was used to identify NK cells. All cytokine gates were drawn on the NK population depicted at left. (G) Flow cytometry gating scheme used to quantitate monocytic THP-1 ADCP is shown for representative serum and plasma samples collected at baseline (left) and month 12.7 (right) from a vaccinated animal (6127). (H) Flow cytometry gating scheme used to quantitate ADNP using human leukocytes is shown for representative serum and plasma samples. Sequential gating (left to right) was used to identify neutrophils, followed by histogram gating of bead-positive neutrophils. Baseline (left) and month 12.7 (right) histograms depict a vaccinated animal (6129). (TIF) [file ppat.1008764.s008.tif]

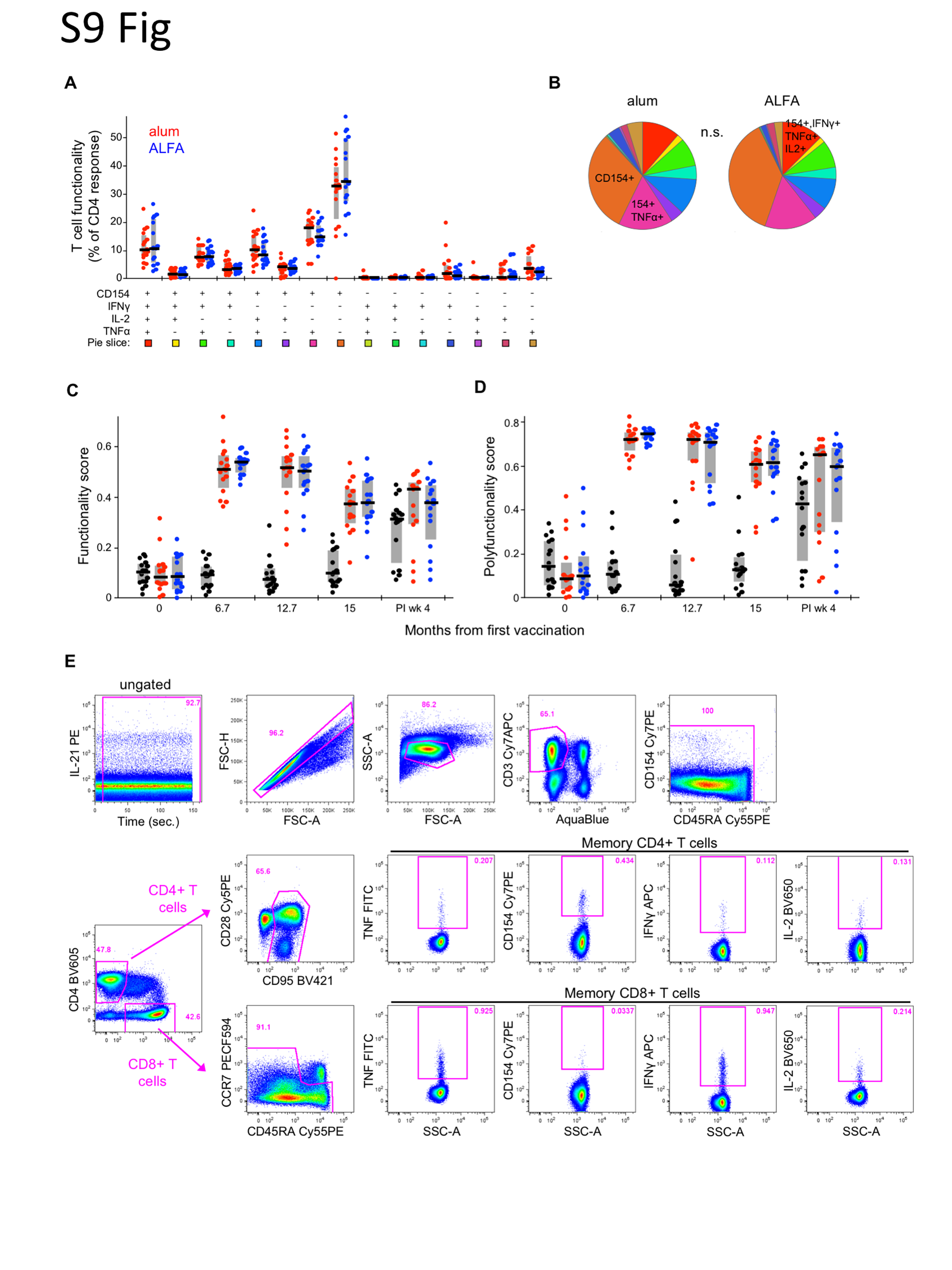

Supplement: S9 Fig — Vaccine-elicited PBMC HIV-1 Env-specific CD4 T cells were measured by ICS for IFNγ, TNFα, IL-2, and CD154 following ex vivo Env PTE peptide pool stimulation. (A-B) Response functionality was assessed by Boolean cytokine combinations represented within the HIV-specific response following the third immunization (month 6 + 3 wks) using SPICE. Bar graphs depict the fraction of the response comprised by each Boolean combination for each animal grouped by vaccine arm; pie charts show the average for each active arm. Predominant Boolean combinations are annotated in the pie charts for reference. (C-D) Longitudinal PBMC CD4 T cell functionality and polyfunctionality scores assessed by COMPASS through week 4 post-infection (PI). Gray bars depict interquartile range; black lines depict medians; No significant differences between active arms were observed. (E) Flow cytometry gating scheme used to identify and quantitate antigen-specific T cell in PBMC is shown from a representative sample (animal 6306, month 12.7, Env PTE pool 3). Sequential gating (top row, left to right) was used to identify T lymphocytes, followed by memory CD4 (middle row) and CD8 (bottom row) gating. All cytokine gates shown were performed with SSC-A on the x-axis. (TIF) [file ppat.1008764.s009.tif]

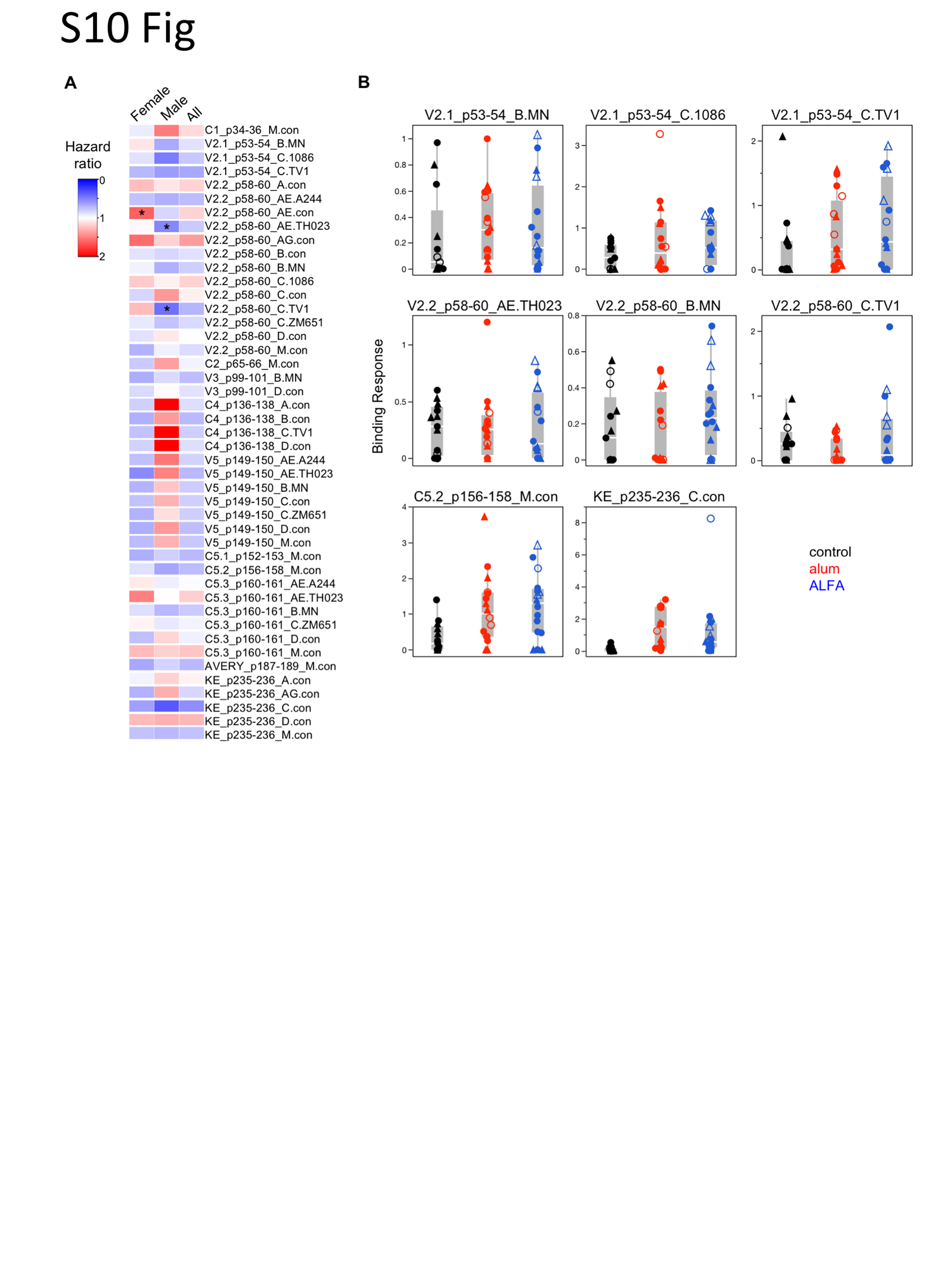

Supplement: S10 Fig — HIV-1 envelope-specific IgG responses were mapped by peptide microarray for rectal secretions at peak immunogenicity (month 12.5). (A) Heatmap depicts association between peak rectal epitope-specific responses and infection risk. Hazard ratios for all peptide array epitope responses that remained in the analysis set after filtering out highly correlated responses are shown, separately by sex and in all animals (*, p<0.05). (B) Epitope-specific binding responses that were protective in males with p<0.2 are shown for each animal by vaccine arm. Outlier box plots depict median, interquartile range, and +/- 1.5 the interquartile range. Female animals are depicted by circles, male by triangles. Open symbols reflect animals that remained uninfected following the ten prespecified challenges. (TIF) [file ppat.1008764.s010.tif]
