# Supplementary material for: Adjuvanted HIV-1 vaccine promotes antibody-dependent phagocytic responses and protects against heterologous SHIV challenge
Source: PLoS Pathog. 2020 Sep 3;16(9):e1008764. doi: 10.1371/journal.ppat.1008764 (PMC7505435; doi:10.1371/journal.ppat.1008764)
Supplement: S1 File — (DOCX) [file ppat.1008764.s017.docx]

**Supplementary Methods**

**Microbiome analysis of lower gastrointestinal tract.** Bacteria were isolated from rectal swabs collected at study month 12 by centrifugation and resuspension in 200 μl of lysis buffer (30 mM Tris-HCl, 10 mM EDTA, 200 mM sucrose, pH 8.2). Samples were heated at 65°C for 10 minutes prior the addition of 10 mg/ml chicken egg white lysozyme solution. Samples were incubated for 1 hour at 37°C. 5% SDS was added to a final concentration of 1% w/v and incubated at 56°C for 10 minutes. DNA extractions were then performed using the QIAamp PowerFecal DNA Kit (Qiagen, Hilden, Germany). Extracted DNA was amplified following the Earth Microbiome Protocol for 16S Illumina sequencing utilizing indexing primers to target the V4 region of the 16S SSU rRNA (515F-806R)[55]. Amplicons from triplicate reactions were combined and then quantified using a Quant-iT PicoGreen dsDNA Assay Kit (ThermoFisher Scientific, Waltham, MA, USA). Amplicons were then added in equal concentration to a library pool and cleaned through a QIAquick PCR purification column (Qiagen) prior to KAPA library quantification (Kapa Biosystems, Wilmington, MA, USA). The pooled library was sequenced using a 2x150 bp Illumina MiSeq run. 16S sequencing reads were processed using QIIME 2 version 2018.2; taxonomic determination in QIIME 2 utilized the Greengenes 13_8 99% OTUs classifier. Alpha and beta diversity metrics were used to examine differences within and between samples, respectively.  Associations with sex, infecting challenge, and vaccine groups were explored.  Microbial composition diversity differences were assessed using both Bray Curtis and weighted Unifrac analyses. Phylum and Family level statistical analysis compared microbial relative abundances by sex using Student's t-tests as well as Cohen's d for effect size.

**Binding antibody Fc array analysis**. Fc array analysis was performed blinded to vaccine arm as previously described [56, 57]. Briefly, sera was analyzed at a dilution of 1:1000 for detection reagents specific for rhesus IgG (Southern Biotech #6200-09, polyclonal, Lot B0112-YC26B), which was also tested at a 1:500 dilution, and for tetramerized rhesus Fcγ receptor (FcγR2A-2, FcγR2A-3, FcγR2A-4, FcγR2B-1, FcγR3A-1, and FcγR3A-3), and human Fcγ receptor (FcγRIIAH, FcγRIIAR, FcγRIIB, FcγRIIIAF, FcγRIIIAV, FcγRIIIB NA1) detection reagents, whereas the dilution used for detection of IgA (Southern Biotech #2050-09, polyclonal, Lot C5213-XA55X) and C1q was 1:250. A panel of 37 SHIV and HIV-1 antigens was evaluated for sera polyclonal antibody responses. Data collection was performed using Luminex Exponent version 4.2 software.

**Infected cell antibody binding assay.** Serum binding antibody to HIV-1 Env expressed on the surface of infected cells was conducted blinded to study arm using a flow-cytometry-based indirect surface staining according to methods similar to those previously described [58]. Briefly, mock infected and SHIV1157 wild-type infectious molecular clone-infected CEM.NKR_CCR5_ cells were incubated with 1:100 diluted serum samples for 2h at 37°C, then stained with a vital dye (Live/Dead Aqua) to exclude dead cells from analysis. CEM.NKR_CCR5_ cells were obtained through the NIH AIDS Reagent Program, Division of AIDS, NIAID, NIH, from Dr. Alexandra Trkola [51, 59, 60]. Cells were washed, permeabilized using BD Cytofix/Cytoperm solution, washed again, and stained with FITC-conjugated goat-anti-rhesus polyclonal antisera to detect binding of the plasma Ab and RD1-conjugated anti-HIV-1 p24 to identify infected cells. Cells positive for rhesus antibody binding were defined as viable, p24 positive, and FITC positive. Results are reported as percent FITC-positive cells and the FITC MFI among the viable p24-positive events after subtracting background observed with secondary antibody alone from both mock and infected cells. Background in mock samples was subtracted from infected cells followed by baseline binding subtraction.

**Neutralizing antibody responses.** Neutralizing antibody titers were determined using TZM-bl cells (NIH AIDS Reagent Program, #8129) in a high-throughput assay utilizing robotic liquid handling. The following pseudoviruses from the indicated subtype were assessed: MW965 (C), GS015 (C), SF162 (B), TH023 (CRF01_AE), CM235 (CRF01_AE), TZA125 (C), TZBD9/11 (C), CO6980v0c22 (C), BZ167 (B), and murine leukemia virus (MuLV) (nonspecific control). Serum was diluted 1:10 in growth medium and serially diluted using the Biomek NXP liquid handler (Beckman Coulter). Titered serum (12.5 μl/well) was transferred to 384-well culture plates and incubated with an equal volume of pseudovirus for 45 min at 37°C. Cells (3x10^3^/well) with DEAE-dextran (40 μg/ml) were added to each well and incubated for 48 h. Relative light units were detected with the SpectraMax Paradigm Microplate Reader (Molecular Devices) using the Bright-Glo Luciferase Assay System (Promega Corporation). Neutralization dose–response curves were fitted by nonlinear regression using the LabKey Server, and the final titer is reported as the reciprocal of the dilution of serum necessary to achieve 50% neutralization (50% inhibitory dose). Sera breadth and potency scores were calculated to account for varying neutralization sensitivities of pseudoviruses tested, as previously described [61]. Sera breadth was calculated by assigning each serum-virus combination a score of 1 or 0, defined as the ID_50_value above (score of 1) or below (score of 0) the median ID_50_ value for all evaluated sera against that virus. Potency was calculated by dividing the serum-virus combination ID_50_value by the median ID_50_ value for all evaluated sera with the same virus.

**Plasma viral load quantitation.** Plasma viral RNA was isolated using a QIAsymphony Virus/Bacteria Midi kit on the QIAsymphony SP instrument.  Viral RNA from 500 µL plasma was eluted with 60 µL buffer.  All subsequent reactions were performed using the automated PCR setup platform, QIAgility.  25 µL of template RNA was annealed to target-specific reverse primers 5’- CAC TAG GTG TCT CTG CAC TAT CTG TTT TG -3’ and reverse transcribed using SuperScript III RT (ThermoFisher, 18080044), PCR Nucleotide Mix (Sigma-Aldrich, 11814362001), and RNaseOUT (ThermoFisher, 10777019) using an optimized version of the manufacturer’s protocol.  Resulting cDNA was treated with RNAse H (ThermoFisher, 18021071) per the manufacturer’s protocol.  10 µL cDNA template was subjected to real-time PCR using TaqMan Gene Expression Master Mix (Applied Biosystems, 4369510) and the target-specific labeled probe 5’- /56-FAM/CTT CCT CAG TGT GTT TCA CTT TCT CTT CTG CG/3BHQ_1/ -3’ and forward 5’- GTC TGC GTC ATC TGG TGC ATT C -3’ and reverse primers 5’- CAC TAG GTG TCT CTG CAC TAT CTG TTT TG -3’ (custom synthesis by Integrated DNA Technologies) on a StepOnePlus Real-Time PCR System (Applied Biosystems) using the standard curve protocol. A standard curve was generated by serial dilution of SIVmac239 *gag* RNA transcribed from a pSP72 vector containing the first 731 bp of *gag* using the Megascript T7 kit (Ambion Inc), quantitated by optical density.  The quality of the RNA standard was assessed using an Agilent Bioanalyzer with RNA Nano 6000 chips (Agilent Inc.).

**Immune correlates sensitivity analysis.** The sensitivity analyses were performed to assess the robustness of the results from the primary analysis: (1) to examine the influence of outliers, the primary analysis was repeated after setting observations more than 3 SD above or below the mean to those thresholds, for a qualitative comparison; (2) the models were fit within the vaccinated animals only, to focus on vaccine-induced immune responses (**S4-S5 Tables**); (3) a similar analysis as (2) but without adjustment for study arm (**S5 Table**), in case part of the effect of an immune response induced by a particular vaccine regimen would be absorbed by that adjustment. The proportional hazards assumption was tested for the sex-specific and combined-sex models, and the sensitivity analyses were used to aid in interpretation when that assumption was found to be violated for a given assay.

Another sensitivity analysis was performed to assess the imputation method used to estimate mucosal peptide-specific IgG binding activity from the epitope microarray. The image intensity for each peptide was analyzed via Cox models, adjusting for baseline intensity, pre- and post-vaccination total IgG, and sex. The results from this analysis were qualitatively compared to those in the primary analysis at the epitope-level, to determine if those results were largely determined by the imputation method used. The sensitivity analysis at the peptide level showed similar protective responses, indicating the results are not likely due to the imputation method used.

**Exploratory Immune Correlates Analyses.** To determine vaccine-induced immune responses, assays were compared between arms via Wilcoxon rank-sum tests, across all animals and within each sex. Additionally, associations between immune responses (at 15 months or the last available time point prior to the challenge) and peak viremia were assessed within the animals infected within 10 challenges, via Spearman correlation and linear models that adjusted for the time of infection (to account for the time between each assay and peak viremia), vaccine arm, and sex. Possible multi-assay associations with the risk of infection were assessed by including all assays in penalized Cox models, separately at 12.5 and 15 months (**S6 Table**). Vaccine arm and sex were included as potential covariates, but assay-by-sex interactions were not included to limit the complexity of the starting model. The optimal penalty was determined via leave-one-out cross-validation, and only protective assays were considered by constraining log hazard ratios to be less than or equal to zero.
